# A Novel Solid Form of Erlotinib: Synthesis by Heterogeneous Complexation and Characterization by NMR Crystallography

**DOI:** 10.1021/acs.cgd.5c00268

**Published:** 2025-04-29

**Authors:** Sean T. Holmes, Ren A. Wiscons, Kerrigan Parks, Sarah Nickel, Halie S. Ankeny, Aaron M. Viggiano, Derek Bedillion, Deben Shoup, Robbie J. Iuliucci, Qiang Wang, Robert W. Schurko, Rosalynn Quiñones

**Affiliations:** † Department of Chemistry & Biochemistry, Florida State University, Tallahassee, Florida 32306, United States; ‡ National High Magnetic Field Laboratory, Tallahassee, Florida 32310, United States; § Department of Chemistry, Amherst College, Amherst, Massachusetts 01002, United States; ∥ Department of Chemistry, Marshall University, Huntington, West Virginia 25755, United States; ⊥ Department of Chemistry, Washington & Jefferson College, Washington, Pennsylvania 15301, United States; # Shared Research Facilities, West Virginia University, Morgantown, West Virginia 25606, United States

## Abstract

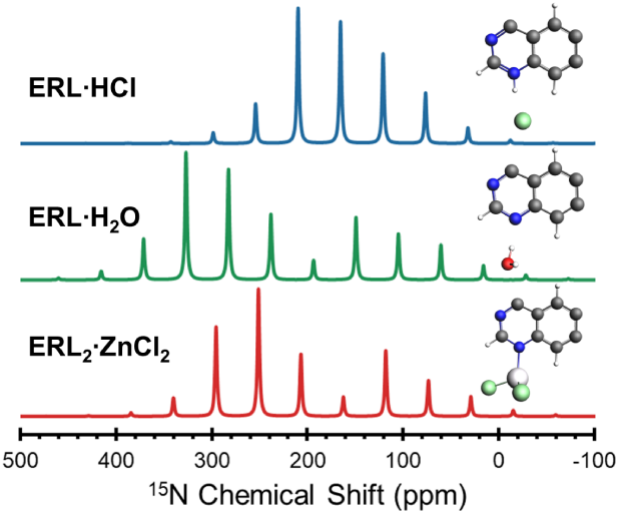

We describe the synthesis of a novel complex of the anticancer "active pharmaceutical ingredient erlotinib (**ERL**) via heterogeneous nucleation on polished zinc tiles. The resulting product, **ERL**_**2**_**·ZnCl**_**2**_, is characterized by single-crystal X-ray diffraction, multinuclear solid-state NMR (ssNMR) spectroscopy, and density functional theory (DFT) calculations. Also characterized are the hydrochloride salt (**ERL·HCl**) and monohydrate free base (**ERL·H**_**2**_**O**) forms of erlotinib. ^13^C ssNMR spectroscopy is useful for site-by-site assignment and rapid fingerprinting, while also providing preliminary structural interpretations, such as the number of molecules in the asymmetric unit. ^35^Cl ssNMR can readily differentiate between the chloride ions in **ERL·HCl** and the covalently bonded chlorine in **ERL**_**2**_**·ZnCl**_**2**_. ^15^N ssNMR proves to be critical here because of the large isotropic chemical shift differences between **ERL·H**_**2**_**O**, **ERL·HCl**, and **ERL**_**2**_**·ZnCl**_**2**_. The ^15^N chemical shift tensors are linked directly to differences in structure and bonding with the aid of DFT calculations. Together, these results demonstrate the utility of multinuclear NMR crystallography for the characterization of solid forms of APIs, especially when other analytical techniques face significant challenges.

## Abstract 2

This paper describes the synthesis of a novel zinc complex of the pharmaceutical erlotinib and the characterization of three solid forms of erlotinib by multinuclear solid-state NMR.

## Introduction

To optimize the physicochemical properties of active pharmaceutical ingredients (APIs), it is essential to explore a diverse array of solid forms, including polymorphs, salts, hydrates, solvates, cocrystals, solid solutions, and amorphous phases.^1−5^ Intermolecular interactions (e.g., hydrogen bonding) within a specific solid form significantly influence its properties, including stability, solubility, dissolution rate, and bioavailability. Therefore, understanding the interplay between structure, properties, and pharmacological function is crucial for the rational design of improved drugs.^6−10^ However, no universally reliable method exists to generate all of the potential solid forms of an API, making the process inherently *ad hoc* and time-intensive. This challenge is further compounded when analytical and/or spectroscopic techniques fail to reliably distinguish between existing solid forms or confirm the discovery of a novel form.

**Scheme 1:**
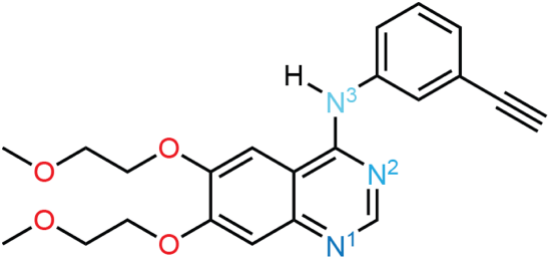
Skeletal Representation of Neutral Erlotinib, with the Numbered Nitrogen Atoms Depicted in Various Shades of Blue to Indicate Qualitative Relative Lewis Basicity; N^1^ is the most Basic and N^3^ is the Least Basic

Due to its promise in treating advanced nonsmall cell lung cancer, erlotinib^11^ (*N*-(3-ethynylphenyl)-6,7-bis(2-methoxyethoxy)-4-quinazolinamine (**ERL**, Scheme 1)) is included in the list of essential medicines by the World Health Organization.^12^ Erlotinib has garnered significant attention for its medicinal importance, the complexity of synthesizing and characterizing its multiple solid forms, and the associated challenges related to safety and intellectual property. Both free-base^13^ and hydrochloride (HCl) salts^14^ of erlotinib, as well as their respective hydrates,^15^ exhibit polymorphism.^16−22^ Additionally, salts (including their polymorphs),^23−26^ cocrystals,^15,26^ amorphous forms,^27^ micro- and nanoparticles,^28,29^ and solid dispersions,^27,29−31^ have been reported, leading to a complicated landscape of solid forms, most of which have not been structurally characterized. There are also questions regarding optimal synthetic protocols for producing these solid forms, including their reproducibility and the purities of the resulting products. All known forms of erlotinib have very low aqueous solubilities (i.e., erlotinib is listed as a BCS Class 2 drug),^32−34^ which decrease their bioavailability and limit their efficacy in clinical applications. Significant effort has been expended preparing formulations with improved solubility and bioavailability, sometimes involving chemical modification of the erlotinib molecule (e.g., through complexation with phospholipids).^29−31,35−40^ Thus, the discovery of novel solid forms of erlotinib, as well as their characterization, could prove fruitful for producing drug formulations with enhanced physicochemical properties.

The variety of erlotinib solid forms is thought to arise, in part, because the N^1^ nitrogen atom (Scheme 1) acts as a strong Lewis base: this explains the protonation at N^1^ for HCl and oxalate salts,^23,26^ as well as why this site serves as a hydrogen-bond acceptor in neutral cocrystals^15,26^ and hydrates.^26^ In nonprotonated forms, the lone pair on N^1^ may also facilitate coordination with metals, as has been observed for metal complexes containing quinazoline or quinoline moieties, which have found applications in medicine, catalysis, optical devices, and other areas.^41−44^ To our knowledge, no metal, transition metal, or post-transition metal complexes of erlotinib have been reported—this is surprising, since such complexes may offer advantageous physicochemical properties in comparison to other solid forms, due to their unique electronic and stereochemical properties.

Solid-state NMR (ssNMR) spectroscopy is a powerful tool for characterizing solid forms of APIs since it provides site-specific information on molecular-level structure and bonding;^45−50^ as such, it can greatly augment structural determination, providing unique insights into hydrogen atom positions and hydrogen bonding that may not be available from single-crystal X-ray diffraction experiments.^51,52^ NMR crystallography (NMRX) techniques, which feature the pairing of ssNMR with quantum chemical calculations using density functional theory (DFT), provide chemical information that is invaluable for the characterization of API solid forms.^53−60^ Furthermore, there are cases in which ssNMR can play a key role in the spectral fingerprinting of different solid forms of APIs, optimization of synthetic protocols, identification of products and unreacted educts, and interpretation of structural features in the absence of diffraction data.^46,48^ In this respect, 1D and/or 2D ssNMR experiments involving ^1^H, ^13^C, and ^15^N are the most common for characterizing APIs. However, quadrupolar nuclides (nuclear spin, *I* > 1/2) such as ^35^Cl also have great potential application here, since their ssNMR spectra are extremely sensitive to long-range hydrogen bonding interactions that are often not revealed from chemical shift measurements.^53,61−64^ To date, NMRX studies have not been carried out on any solid form of erlotinib; however, for the reasons mentioned above, ssNMR methods are expected to be of great utility for these systems.

In light of this, herein we describe the preparation of a new zinc complex of erlotinib, **ERL**_**2**_**·ZnCl**_**2**_, using heterogeneous nucleation on zinc tiles, as well as its structural characterization using single-crystal X-ray diffraction (SCXRD), X-ray photoelectron spectroscopy (XPS), powder X-ray diffraction (PXRD), differential scanning calorimetry (DSC), infrared spectroscopy, ssNMR **s**pectroscopy, and DFT calculations. Two other solid forms of erlotinib, including its monohydrate free base (**ERL·H**_**2**_**O**) and hydrochloride salt (**ERL·HCl**), were also produced and characterized by these methods. Only **ERL·H**_**2**_**O** has a known crystal structure,^26^ whereas **ERL·HCl** is referred to as “Form B” in the patent literature.^25^ Additionally, attempts were made to isolate and characterize an anhydrous free-base form of erlotinib, **ERL·FB**. We highlight the use of multinuclear ssNMR for the characterization of these solid forms, since it provides insights into structure and bonding that is not available from other techniques.

## Experimental and Computational Details

### Materials

**ERL·HCl** was purchased from ApexBio Tech (>99%), whereas **ERL·FB** was purchased from LC Laboratories (>99%). **ERL·H**_**2**_**O** was obtained by recrystallizing **ERL·FB** from H_2_O.^26^**ERL**_**2**_**·ZnCl**_**2**_ was prepared by recrystallization on Zn tiles (Goodfellow Cambridge Ltd., 99.95+%) that were sanded, cleaned, and cut into 1 cm^2^ squares. A 16 mM **ERL·HCl** solution in methanol was prepared using sonication and heat. The Zn tiles were placed into well plates and 500 μL of the **ERL·HCl** solution was added to each. The solution was left to evaporate slowly at room temperature by covering it with aluminum foil. Following solvent evaporation, the crystals that formed at the top of each tile were carefully removed for further analysis.

### Single-Crystal X-Ray Diffraction

SCXRD images were collected using a Rigaku XtaLAB Synergy-i X-ray diffractometer configured in a kappa goniometer geometry. The diffractometer, which is equipped with a low-temperature device and a PhotonJet-S microfocus Cu source (λ = 1.54187 Å), was operated at 50 kV and 1 mA. X-ray intensities were measured at 150 K with the Bantam detector placed 44.00 mm from the sample. The data were processed with CrysAlisPro version 41_64.117a (Rigaku Oxford Diffraction) and corrected for absorption. The structures were determined in OLEX2^65^ using SHELXT^66^ and refined using SHELXL.^67^ All non-hydrogen atoms were refined anisotropically with hydrogen atoms placed at idealized positions except in cases of significant positional disorder. Single crystals were mounted on a 150 μm MiTeGen MicroMount using mineral oil.

### Powder X-Ray Diffraction

PXRD patterns were collected using a Rigaku XtaLAB Synergy-i X-ray diffractometer configured in a kappa goniometer geometry. The diffractometer is equipped with a low-temperature device and a PhotonJet-S microfocus Cu source (λ = 1.54187 Å) and operated at 50 kV and 1 mA. X-ray intensities were measured at room temperature with the Bantam detector placed 44.00 mm from the sample. All images were collected with a continuous ϕ-rotation scan with a 200 s exposure time. Due to the instrument configuration, minimal sample sizes were required (sufficient sample to pack into a sphere 150–200 μm in diameter). This data collection strategy also minimizes the effects of preferred orientation. The images were processed into diffraction patterns using CrysAlisPro version 41_64.117a (Rigaku Oxford Diffraction). Samples were mounted on a 150 μm MiTeGen MicroMount using mineral oil. Additional PXRD patterns were obtained using a PANalytical X’Pert Pro MPD powder X-ray diffractometer with Cu *K*α X-ray source operating at 45 kV and 40 mA power in the Bragg–Brentano geometry. The patterns were collected over a 2θ range of 5° to 80° at a step size of 0.017° with a solid-state X-ray detector.

### Solid-State NMR Spectroscopy

ssNMR experiments at *B*_0_ = 9.4 T were conducted using a Varian Inova spectrometer and Oxford magnet, with Larmor frequencies of ν_0_(^1^H) = 399.8 MHz, ν_0_(^13^C) = 100.5 MHz, and ν_0_(^15^N) = 40.5 MHz. High-field ^35^Cl ssNMR experiments were conducted at the National High Magnetic Field Laboratory using a Bruker Avance NEO console and a home-built 21.1 T ultrawide bore magnet,^68^ with Larmor frequencies of ν_0_(^1^H) = 894.52 MHz and ν_0_(^35^Cl) = 87.64 MHz. Additional experiments were conducted at 18.8 T using a Bruker Avance III spectrometer with Larmor frequencies of ν_0_(^1^H) = 800.1 MHz and ν_0_(^35^Cl) = 78.4 MHz. All ssNMR spectra were processed and analyzed using the ssNake v1.4 software package.^69^ Further details of the ssNMR experiments are provided in Supplement S1. All pulse sequences and recommended calibration parameters and standards are available from the authors by request or at https://github.com/rschurko.

### Quantum Chemical Calculations

All quantum chemical calculations were performed using DFT methods. Refinements of crystal structures were performed using the CASTEP module within Materials Studio 2020.^70^ Calculations of NMR interaction tensors were performed using Amsterdam Density Functional 2021.^71^ Details of these calculations are provided in Supplement S2.

## Results and Discussion

### Synthesis and Characterization of ERL_2_·ZnCl_2_

A 16 mM solution of **ERL·HCl** in methanol was placed on polished square zinc tiles with a surface area of 1 cm^2^ and allowed to evaporate under ambient conditions. This yielded white crystals on the surface that were carefully removed for further analysis. It was initially assumed that the novel form prepared in this manner was simply another polymorph, hydrate, or salt of erlotinib, adding to the abundance of known solid forms. However, it initially proved difficult to obtain suitable crystals for analysis by SCXRD. Instead, other analytical characterization methods were pursued.

The white crystals were initially characterized by solution NMR, DSC, and XPS. ^1^H and ^13^C NMR spectra of the sample dissolved in DMSO-*d*_6_ indicate the presence of the free base, via comparison to analogous spectra of **ERL·H**_**2**_**O** and **ERL·HCl** (Figure S5a,b). DSC scans revealed thermal behavior more akin to that of **ERL·HCl** than **ERL·H**_**2**_**O** (Supplement S3). XPS revealed the presence of zinc and chlorine, suggesting the potential formation of a zinc complex (Supplement S4). Infrared spectra were also acquired, which are useful for fingerprinting (Supplement S5). Collectively, these data encouraged further analysis of the sample, as described below.

### Single-Crystal X-Ray Diffraction

**Figure 1:**
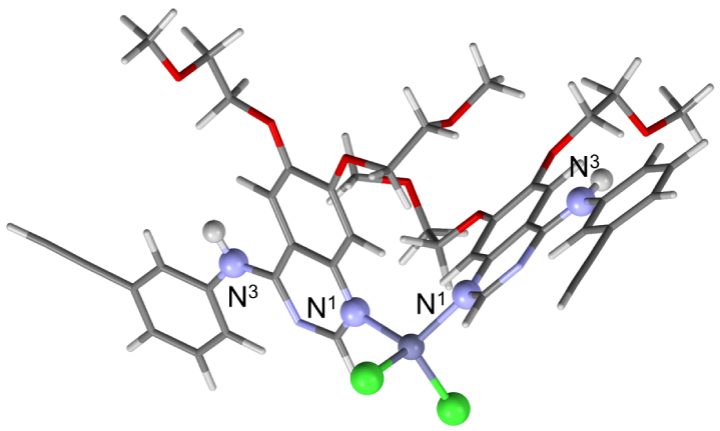
Crystal structure of **ERL_2_·ZnCl_2_** viewed along the crystallographic *b*-axis, as determined by single-crystal X-ray diffraction and subsequent plane-wave DFT geometry optimization. The nitrogen atoms N^1^ and N^3^ are indicated.

Selection of the best crystals from the sample led to the determination of the structure of a novel complex, **ERL**_**2**_**·ZnCl**_**2**_, using SCXRD at 150 K (Figure 1, Table 1). The crystal structure is available from the Cambridge Structural Database under deposition number 2403615. ORTEP images of the crystal structure are provided in Figure S6. **ERL**_**2**_**·ZnCl**_**2**_ crystallizes in the *P*2_1_/*n* space group with *Z*′ = 1 and *Z* = 4, and features a pseudotetrahedral Zn center bound to two Cl atoms and two molecules of erlotinib through N^1^. Zn–Cl bond lengths, *r*(Zn···Cl), of 2.275 Å and 2.276 Å (DFT geometry optimized), are slightly shorter than those of ZnCl_2_ (2.304 Å).^72^ There are two N^3^–H···Cl hydrogen bonds with distances, *r*(N^3^–H···Cl), of 2.558 and 2.584 Å (geometry optimized structure). Finally, there is some degree of disorder evident in two of the four of the methoxy moieties.

**Table 1:** Single-Crystal X-Ray Diffraction Data and Refinement Parameters for **ELR_2_·ZnCl_2_**

Crystal Data	
Chemical formula	C_44_H_46_N_6_O_8_ZnCl_2_
*M*_r_ (amu)	923.16
Crystal system, space group	Monoclinic, P2_1_/n
Temperature (K)	150(1)
*a*, *b*, *c* (Å)	11.2148 (7), 23.9974 (13), 16.2008 (9)
β (°)	100.221
*V* (Å^3^)	4290.87
*Z*	4
Radiation type	Cu Kα
Crystal size (mm)	0.028 × 0.054 × 0.115
Data collection	
Diffractometer	XtaLAB Synergy, Single source at home/near, HyPix3000
Absorption correction	Multiscan CrysAlis PRO 1.171.41.117a (Rigaku Oxford Diffraction, 2021) empirical absorption correction using spherical harmonics, implemented in SCALE3 ABSPACK scaling algorithm.
Tmin, Tmax	0.945, 1.000
No. of measured, independent, and observed [*I* > 2σ(*I*)] reflections	22327, 7696, 4859
Rint	0.061
(sin θ/λ)_max_ (Å^–1^)	0.603
Refinement Information	
*R*[*F*^2^ > 2σ(*F*^2^)], w*R*(F^2^), *S*	0.0643, 0.1707, 1.037
No. of reflections	7696
No. of parameters	575
H atom treatment	H atoms treated by a mixture of independent and constrained refinement
Δρ_max_, Δρ_min_ (e Å^–3^)	0.54, −0.62

### Powder X-Ray Diffraction

**Figure 2:**
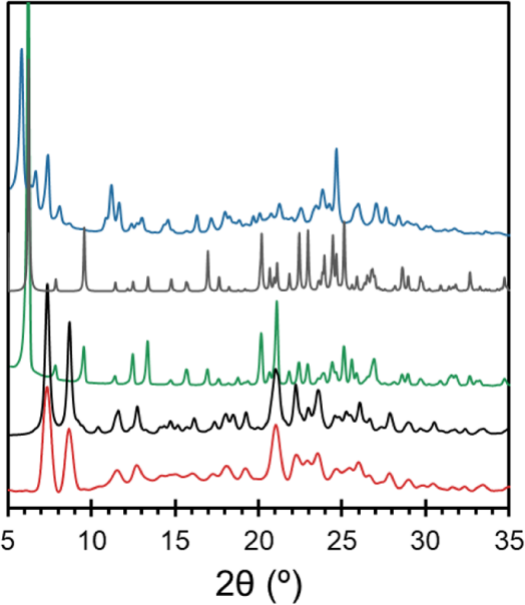
Powder X-ray diffraction patterns for three solid forms of erlotinib, including **ERL·HCl** (blue), **ERL·H_2_O** (green), and **ERL_2_·ZnCl_2_** (red). Simulated patterns based on the known crystal structures of **ERL·H_2_O** and **ERL_2_·ZnCl_2_** are shown in gray and black, respectively.

PXRD (Figure 2) was used to investigate the purity of the **ERL**_**2**_**·ZnCl**_**2**_ samples crystallized via the heterogeneous nucleation method. Given the small sample size, PXRD patterns were extracted from continuous φ-rotation diffraction images collected using a SCXRD instrument, revealing that **ERL**_**2**_**·ZnCl**_**2**_ exists uniformly throughout the bulk microcrystalline sample, as confirmed by comparison to a simulated PXRD pattern based on the new crystal structure. **ERL·H**_**2**_**O** was demonstrated to be pure by similar means,^23,26^ whereas PXRD provides a fingerprint for **ERL·HCl**.

### Solid State NMR Spectroscopy

In this section, we discuss the application of ^13^C, ^15^N, and ^35^Cl ssNMR for the structural characterization and spectral fingerprinting of **ERL**_**2**_**·ZnCl**_**2**_, **ERL·H**_**2**_**O**, and **ERL·HCl**. Quantum chemical calculations are used to provide site assignments and assess the impact of molecular-level structure and bonding on chemical shift and electric field gradient (EFG) tensors.^73^

**Figure 3:**
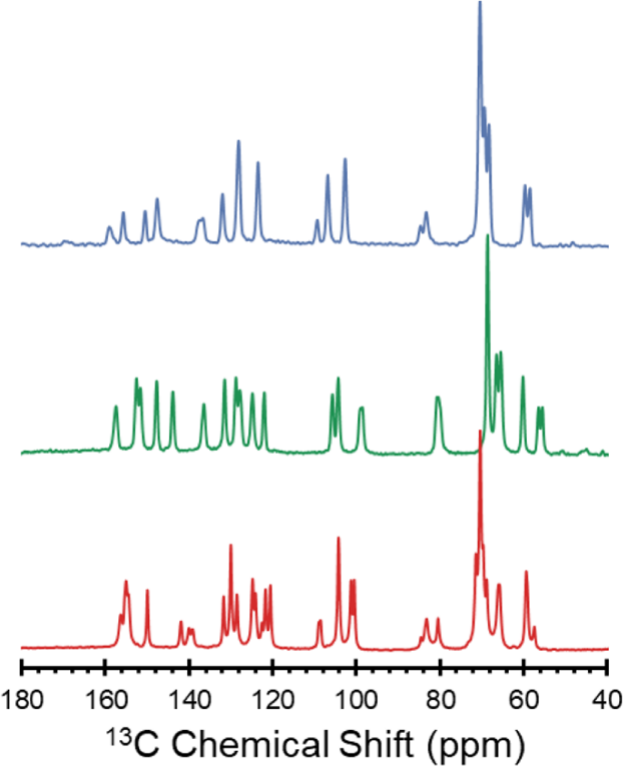
^1^H–^13^C CP/MAS spectra (ν_rot_ = 10–12 kHz) of **ERL·HCl** (blue), **ERL·H_2_O** (green), and **ERL_2_·ZnCl_2_** (red) acquired at 9.4 T.

^1^H–^13^C CP/MAS spectra reveal significant differences between each solid form (Figure 3). The number of peaks in the spectrum of **ERL**_**2**_**·ZnCl**_**2**_ is greater than the expected 22 peaks due to the presence of two erlotinib molecules in the asymmetric unit. Differences are evident in the ^13^C chemical shifts associated with the quinazoline groups due to variations in their structure and bonding at the N^1^ sites. The ^13^C chemical shifts of the ethynylphenyl ring are similarly influenced. Assignments of ^13^C peaks were assisted by DFT calculations of isotropic chemical shifts; however, these are only approximate, especially when differences between chemical shifts are very small (i.e., less than 2 ppm): a summary of computational results is given in Figure S7. We note that ^1^H–^13^C CP/MAS experiments may be a viable pathway for monitoring the interconversion between solid forms *in situ*. For example, our attempts to isolate a pure phase of **ERL·FB** were hindered by its conversion to **ERL·H**_**2**_**O**—this process was monitored *in situ* for a sample of **ERL·FB** packed in a 4.0 mm o.d. rotor over 48 h, demonstrating partial conversion to **ERL·H**_**2**_**O** (Figure S8).

**Figure 4:**
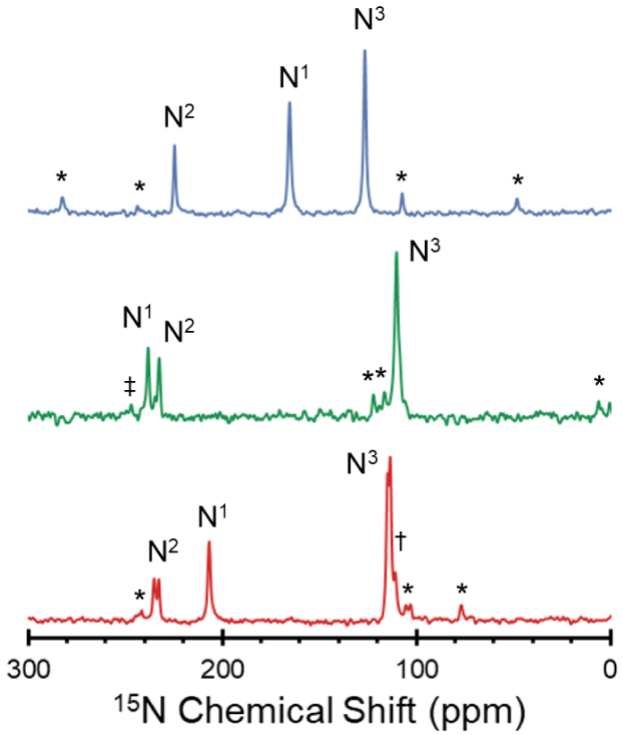
High resolution ^1^H–^15^N CP/MAS spectra (ν_rot_ = 4.70 – 5.25 kHz) of **ERL·HCl** (blue), **ERL·H_2_O** (green), and **ERL_2_·ZnCl_2_** (red) acquired at 9.4 T. Spinning sidebands are marked with an asterisks. The spectrum for **ERL_2_·ZnCl_2_** shows a doubling of the N^2^ and N^3^ peaks due to the presence of two molecules in the asymmetric unit. A small amount of an unidentified impurity phase appears in the baseline of the spectrum for **ERL_2_·ZnCl_2_** next to the N^3^ peak (†). There is also a small impurity in the spectrum of spectrum for **ERL·H_2_O** (‡) near the N^1^ peak, likely corresponding to a polymorph or an anhydrous phase of ERL.

Differences in structure and bonding between the solid forms of erlotinib are prominently reflected in variations of the ^15^N isotropic chemical shifts measured from the ^1^H–^15^N CP/MAS NMR spectra, collected at natural abundance (n.a.(^15^N) = 0.368%, Figure 4). The spectra for **ERL·H**_**2**_**O** and **ERL·HCl** each feature three peaks, whereas that of **ERL**_**2**_**·ZnCl**_**2**_ features six peaks due to the presence of two ERL molecules in the complex (*N.B.*: splitting of the N^1^ peaks is only *ca*. 20 Hz). **ERL·HCl**, which is protonated at the N^1^ site, has δ_iso_(N^1^) = 165.2 ppm, whereas the nonprotonated **ERL·H**_**2**_**O** has δ_iso_(N^1^) = 238.0 ppm. Between these extremes, **ERL**_**2**_**·ZnCl**_**2**_, which features N^1^ coordination with Zn, has δ_iso_(N^1^) = 206.7 ppm. The variation in the δ_iso_ for the arylamine nitrogen N^3^ follows the reverse trend (**ERL·HCl** > **ERL**_**2**_**·ZnCl**_**2**_ > **ERL·H**_**2**_**O**), although the differences are smaller (*ca*. 16 ppm)—this is unsurprising since N^3^ is not within a conjugated heterocycle. In contrast, there is less variability in the values of δ_iso_(N^2^) (a range of *ca*. 10 ppm), in part because this site is not involved in hydrogen bonding or zinc coordination.

**Figure 5:**
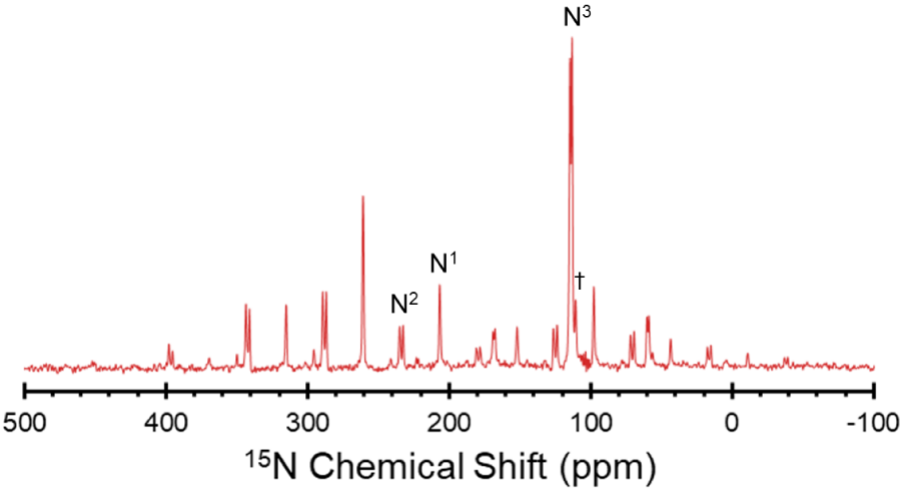
Slow-spinning ^1^H–^15^N CP/MAS (ν_rot_ = 2.2 kHz) spectrum of **ERL_2_·ZnCl_2_** (red) acquired at 9.4 T. A small amount of an unidentified impurity phase appears in the baseline of the spectrum next to the N^3^ peak (†).

The nitrogen chemical shift tensors, obtained from ^1^H–^15^N CP/MAS NMR spectra obtained under conditions of slow MAS (Figure 5, Table 2 and Figure S9a–c), reflect the electronic structures of the erlotinib molecules in each solid form to a greater degree than the isotropic shifts. The large variations in δ_iso_(N^1^) in the different bonding environments are due to differences in chemical shift anisotropy (CSA) that stem from disparities in all three principal components, with δ_11_ and δ_22_ exhibiting the largest differences; notably, δ_22_ varies by as much as *ca*. 150 ppm between **ERL·H**_**2**_**O** (331 ppm) and **ERL·HCl** (184 ppm), with **ERL**_**2**_**·ZnCl**_**2**_ falling between these (290 ppm). The ^15^N chemical shift tensors for the arylamine N^3^ sites also reflect changes in electronic structure due to the bonding environment of N^1^. In part, this is because the arylamine lone-pair electrons are delocalized in the quinazoline ring, as evidenced by the fact that the arylamine is coplanar with the quinazoline ring. However, because the chemical shift span of the arylamines is smaller than that observed for aromatic nitrogen atoms N^1^ and N^2^, the variation is less pronounced. The most significant change is again observed for δ_11_, where the difference between **ERL·HCl** and the **ERL·H**_**2**_**O** is 38 ppm. As anticipated, the value of δ_11_ for **ERL**_**2**_**·ZnCl**_**2**_ falls between these. The variation in the principal values of N^2^ between the different forms is comparatively modest.

**Table 2:** Experimental ^15^N Chemical Shift Tensors for Three Solid Forms of ERL^a^^b^^c^

		δ_iso_ (ppm)	δ_11_ (ppm)	δ_22_ (ppm)	δ_33_ (ppm)	Ω (ppm)	κ
**N1**							
ERL·HCl		165.2	276(3)	184(2)	36(2)	240	0.24
ERL·H_2_O		238.0	380(8)	331(6)	3(5)	377	0.74
ERL_2_·ZnCl_2_	Avg. M1 and M2^d^	206.7	326(7)	290(5)	5(5)	321	0.78
**N2**							
ERL·HCl		224.6	396(5)	299(4)	–21(3)	417	0.54
ERL·H_2_O		232.4	405(5)	305(4)	–12(3)	417	0.52
ERL_2_·ZnCl_2_	M1	232.7	416(14)	305(10)	–23(9)	439	0.49
	M2	235.0	421(6)	311(4)	–26(4)	447	0.51
**N3**							
ERL·HCl		126.5	201(2)	117(2)	62(2)	139	–0.21
ERL·H_2_O		110.1	163(2)	97(2)	70(2)	93	–0.42
ERL_2_·ZnCl_2_	M1	113.3	184(2)	100(2)	56(2)	128	–0.31
	M2	114.8	186(2)	100(2)	59(2)	127	–0.35

a Theoretical chemical shift tensors were obtained from calculations on XRD-derived structural models refined using dispersion-corrected plane-wave DFT calculations. The magnetic shielding tensors were calculated at the PBE0 level.

b The principal components of the chemical shift tensors are defined using the frequency-ordered convention, with δ_11_ ≥ δ_22_ ≥ δ_33_. The isotropic chemical shift, span, and skew are given by δ_iso_ = (δ_11_ + δ_22_ + δ_33_)/3, Ω = δ_11_ – δ_33_, and κ = 3(δ_22_ – δ_iso_)/Ω, respectively.

c The uncertainties in the principal components of the chemical shift tensors (in parentheses) depend on many factors, including signal-to-noise, overlapping peaks, the number of spinning sidebands, and the size of the chemical shift anisotropy. The average uncertainty was estimated to be ca. 4 ppm, with a few extreme values extending over 10 ppm (these large uncertainties correspond to the nitrogen atoms in **ERL_2_·ZnCl_2_** with unresolved peaks in their corresponding spectra).

d These values represent averages for two nitrogen atoms that could not be differentiated.

Nitrogen magnetic shielding tensors can now be calculated with a high degree of accuracy; as such, they are increasingly used for site assignments and interpretation of electronic effects on molecular structure.^58,59,74−77^ Experimental and computed ^15^N chemical shift tensors are summarized in Figure S10. Agreement between calculation and experiment is good, with chemical shift distances (see Supplement S2)^78^ ≤10 ppm for each nitrogen site.

One important insight provided by DFT calculations is the orientation of the principal components of the ^15^N chemical shift tensors with respect to the molecule. This can be demonstrated by considering the orientation of the ^15^N chemical shift tensor for N^1^, since it features the most variability between the three solid forms (Figure S11). In **ERL·H**_**2**_**O**, δ_11_ and δ_22_ reside in plane of the quinazoline ring with δ_22_ pointing in general direction of the N^1^···H hydrogen bonding axis with the water molecule. In **ERL**_**2**_**·ZnCl**_**2**_, δ_11_ and δ_22_ are also in the plane of ring, but with δ_11_ pointing in the general direction of the N^1^–Zn coordinating bonding axis. In both cases, δ_33_ is oriented perpendicular to the ring, which reflects why there is less variability in this principal component. Furthermore, calculations on systems with known structures lend insight into systems with unknown structures, such as **ERL·HCl**. In the protonated species **ERL·HCl**, δ_11_ is likely aligned along (or near) the N^1^–H···Cl^–^ hydrogen bonding axis. From this observation, it is clear that the principal component of the ^15^N chemical shift tensor that is oriented perpendicular to the relevant bonding axis follows the trend **ERL·H**_**2**_**O** (δ_11_ = 380 ppm) > **ERL**_**2**_**·ZnCl**_**2**_ (δ_22_ = 290 ppm) > **ERL·HCl** (δ_22_ = 184 ppm). Analysis of the ^15^N magnetic shielding tensors of the arylamine N^3^ sites provide complementary structural information (Figure S12). For each solid form, δ_33_ is similarly oriented along or near the N–H bonding axis, with δ_22_ approximately within the plane of the molecule, and δ_11_ perpendicular to it. Here, it is again clear that the principal components of the ^15^N chemical shift tensor that are most reflective of differences in bonding are those perpendicular to the bonding axis. For **ERL·HCl**, this further indicates that N^3^ must participate in N^3^–H···Cl^–^ hydrogen bonding.

**Figure 6:**
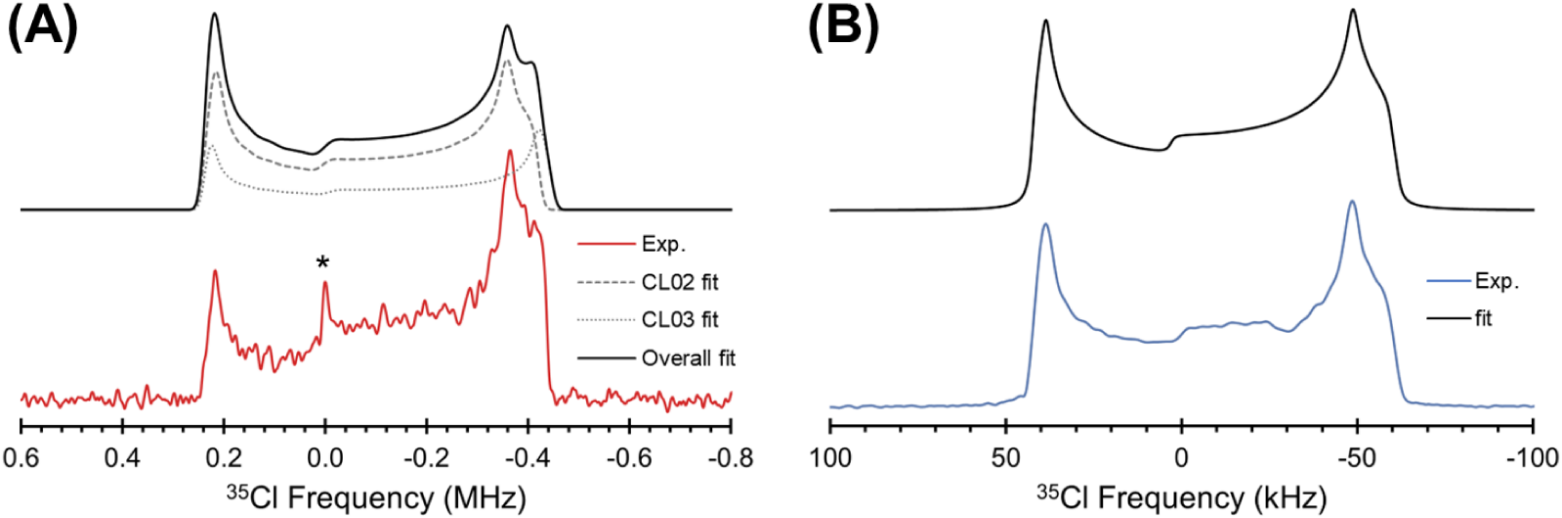
(A) A ^35^Cl ssNMR spectrum of **ERL_2_·ZnCl_2_** acquired at 21.1 T using the WURST-CPMG pulse sequence (red), along with a simulated pattern (black) and a deconvolution of the pattern into the two underlying components, corresponding to the two types of chlorine atoms (CL02 and CL03). The asterisk indicates an additional signal that may correspond to a small amount of **ERL·HCl**, or a polymorph thereof. (B) A ^35^Cl ssNMR spectrum of **ERL·HCl** acquired at 18.8 T using the CPMG pulse sequence (blue), along with a simulated pattern (black).

An ultrawideline ^35^Cl spectrum of **ERL**_**2**_**·ZnCl**_**2**_, acquired at *B*_0_ = 21.1 T (Figure 6A), features a single central-transition (CT, +1/2 ↔ −1/2) powder pattern with a breadth of *ca*. 725 kHz. The spectrum was acquired using the WURST-CPMG pulse sequence,^79,80^ which affords broadband excitation and refocusing of spin polarization, as well as *T*_2_-based signal enhancement, both of which are essential for acquiring spectra with ultrawideline powder patterns, which can range in breadth from 250 kHz to upward of 10 MHz.^81^ Unfortunately, **ERL**_**2**_**·ZnCl**_**2**_ has a small *T*_2_^eff^(^35^Cl) constant, resulting in a short CPMG echo train and correspondingly low signal-to-noise ratio (Figure S13); however, there is enough detail in the spectrum to warrant simulation and analysis.

The ^35^Cl ssNMR spectrum of **ERL**_**2**_**·ZnCl**_**2**_ indicates the presence of two overlapping patterns, corresponding to the two crystallographically and magnetically distinct chlorine atoms (see Figure S14 for additional evidence of this, based on a spectrum with much higher resolution but lower signal-to-noise). The powder patterns are dominated by the second-order quadrupolar interaction, with no clear indication of chemical shift anisotropy. Numerical fits yield the δ_iso_ values, quadrupolar coupling constants (*C*_Q_), and asymmetry parameters (η_Q_), which are summarized in Table 3 along with values derived from DFT calculations. The low values of η_Q_ indicate axially symmetric EFG tensors, which are consistent with chlorine atoms participating in terminal zinc-chlorine bonding. The values of *C*_Q_ are similar to those in previous reports for Cl atoms in transition metal and post-transition metal compounds, which typically fall within the range of *ca*. 10–30 MHz, with the large range of values reflecting the diverse natures of metal–ligand bonds.^82−88^ DFT calculations show that the sign of *C*_Q_ is negative for both chlorine atoms, with the EFG tensor oriented such that its largest principal component, *V*_33_, is approximately along the Zn–Cl bonding axis; therefore, the EFG diminishes along this axis away from the nucleus. From these calculations, we assign the ^35^Cl ssNMR narrower powder pattern (*C*_Q_ = 20.4 MHz) to the crystallographic site CL02, and the broader pattern (*C*_Q_ = 21.2 MHz) to CL03. The differences in the EFG tensors do not appear to be related to the Zn–Cl bond lengths (because these are nearly identical), and likely result from subtle variations in hydrogen bonding with N^3^. The values of *C*_Q_ are slightly smaller than that of the chlorine atom in ZnCl_2_ (*C*_Q_ = 22.10 MHz, Figure S15), possibly indicating that complexation with erlotinib decreases the covalency of the Zn–Cl bonds;^89^ however, the chlorine atoms in ZnCl_2_ reside in a bridging environment (i.e., each is bound to two zinc atoms), resulting in a nonaxial ^35^Cl EFG tensor. (*N.B.*: the analysis of the ^35^Cl EFG tensor of ZnCl_2_ will be the subject of an upcoming study from one of our laboratories.) There is also an additional narrow feature in the pattern (marked by an asterisk) that may be indicative of a small quantity of a chloride salt in the sample.

**Table 3:** Experimental and Calculated ^35^Cl Chemical Shifts and EFG Tensors for the Two Chlorine Atoms in **ERL_2_·ZnCl**_2_ and the Single Chlorine Atom in **ERL·HCl**^a^^b^^c^^d^

Chlorine Site		δ_iso_ (ppm)	C_Q_ (MHz)	η_Q_
ERL_2_·ZnCl_2_ (CL02)	Exp.	50(100)	20.4(3)	0.06(2)
	Calc.	–21	–22.38	0.08
ERL_2_·ZnCl_2_ (CL03)	Exp.	–100(100)	21.2(3)	0.03(2)
	Calc.	–38	–22.84	0.06
ERL·HCl	Exp.	74(5)	7.48(6)	0.13(3)
	Calc.	n/a^e^	n/a^e^	n/a^e^

a Theoretical chemical shifts and EFG tensors were obtained from calculations on XRD-derived structural models refined using dispersion-corrected plane-wave DFT calculations.

b Chemical shifts are reported relative to 0.1 M NaCl. Calculated values are converted to the chemical shift scale by setting the computed shielding for l-histidine HCl·H_2_O to 34.5 ppm.

c The experimental uncertainties in the last digit for each value are indicated in parentheses.

d The principal components of the EFG tensors are defined such that |*V*_33_| ≥ |*V*_22_| ≥ |*V*_11_|. The quadrupolar coupling constant and asymmetry parameter are given by *C*_Q_ = *eQV*_33_/*h*, and η_Q_ = (*V*_11_ – *V*_22_)/*V*_33_, respectively. The sign of *C*_Q_ cannot be determined from the experimental ^35^Cl spectra.

e Since there is not a known crystal structure for **ERL·HCl**, no calculated NMR interaction tensors are reported.

In contrast, the ^35^Cl spectrum of **ERL·HCl** (Figure 6B), obtained at 18.8 T using a CPMG pulse sequence,^90^ reveals a comparatively narrow pattern (*ca*. 105 kHz at 18.8 T), which is consistent with a chloride ion participating only in intermolecular hydrogen bonding, rather than covalent bonding.^61,63,64,91^ The values of *C*_Q_ and η_Q_ for **ERL·HCl** are summarized in Table 3, with a more complete analysis summarized in Table S2. Although the crystal structure of this polymorph is unknown, the high value of *C*_Q_ (7.48 MHz) and low value of η_Q_ (0.13) are consistent with a chloride ion featuring either a single short hydrogen bond (i.e., less than 2.2 Å), or two short contacts separated by *ca*. 180°—the latter configuration of hydrogen bonds is observed in the other known polymorph (as depicted in Figure S16).^23^ Furthermore, the analysis of the ^15^N magnetic shielding tensors of N^1^ and N^3^ in **ERL·HCl** (*vide supra*) suggest that both of these sites participate in N–H···Cl^–^ hydrogen bonding. These insights into the hydrogen bonding network of **ERL·HCl** may prove critical in future crystallographic investigations, possibly involving NMR-guided crystal structure prediction^53,56^ and/or NMR-guided Rietveld refinement methods.^92^

## Conclusions

A novel solid form of the anticancer drug erlotinib was synthesized through complexation on polished zinc tiles. Analysis by single-crystal XRD revealed the formation of a metal-API complex for the form **ERL**_**2**_**·ZnCl**_**2**_. This novel API solid form, along with **ERL·H**_**2**_**O** and **ERL·HCl**, were characterized using a multinuclear NMR crystallography approach. This approach features experimentation on the NMR-active nuclides ^13^C, ^15^N, and ^35^Cl, and proves to be exceptionally useful for the structural characterization of API solid forms that are difficult to analyze through other methods. In addition to providing spectral fingerprints that serve as to unambiguously identify the solid form, these NMR spectra also provide a wealth of chemical information that is linked to crystal structure, hydrogen bonding, and API-metal complexation. In particular, we find that ^15^N chemical shift tensors are exquisitely sensitive to electronic structure resulting from differences in protonation state, intermolecular noncovalent interactions such as hydrogen bonding, and the bonding between nitrogen and zinc atoms. The variation in ^15^N chemical shift tensors was reproduced using state-of-the-art DFT calculations, which provide their key relationships with molecular-level structure and bonding, and even offer significant insight in cases where crystal structures are unknown. Together, these techniques lend insight into the crystal structures and chemical bonding within the solid forms of erlotinib, provide an avenue for the discovery of additional solid forms of erlotinib, suggest future applications to the characterization of a diverse array of solid forms of APIs using multinuclear NMR crystallography, and could aid in improving crystal structure prediction routines for multicomponent API solid forms.

## Supporting Information Available

Additional experimental and computational details; DSC analysis; XPS analysis; IR analysis; solution-state ^1^H and ^13^C NMR analysis; additional details of the crystal structure; additional multinuclear ssNMR analysis (PDF)
