# Supplementary material for: A Novel Solid Form of Erlotinib: Synthesis by Heterogeneous Complexation and Characterization by NMR Crystallography
Source: Cryst Growth Des. 2025 Apr 29;25(9):3219–28. doi: 10.1021/acs.cgd.5c00268 (PMC12063053; doi:10.1021/acs.cgd.5c00268)
Supplement: Supplementary file 1 — cg5c00268_si_001.pdf [file cg5c00268_si_001.pdf]

Supporting Information for:

A Novel Solid Form of Erlotinib: Synthesis by Heterogeneous Complexation and Characterization by NMR Crystallography

Sean T. Holmes,[1,2] Ren A. Wiscons,[3] Kerrigan Parks,[4] Sarah Nickel,[4] Halie S. Ankeny,[5] Aaron M. Viggiano,[5] Derek Bedillion,[5] Deben Shoup,[4] Robbie J. Iuliucci,[5] Qiang Wang,[6] Robert W. Schurko,[1,2,*] Rosalynn Quiñones[4,*]

[1] Department of Chemistry & Biochemistry, Florida State University, Tallahassee, FL 32306
[2] National High Magnetic Field Laboratory, Tallahassee, FL 32310
[3] Department of Chemistry, Amherst College, Amherst, MA 01002
[4] Department of Chemistry, Marshall University, Huntington, WV 25755
[5] Department of Chemistry, Washington & Jefferson College, Washington, PA 15301
[6] Shared Research Facilities, West Virginia University, Morgantown, WV 25606
* Authors to whom correspondence should be addressed
Email addresses: rschurko@fsu.edu, quinonesr@marshall.edu

Supplement S1. Experimental details of solid-state NMR

Overview. Moderate-field ssNMR experiments were conducted at Washington & Jefferson College (Washington, PA) using a Varian Inova spectrometer and 9.4 T Oxford magnet, with corresponding to Larmor frequencies of $\nu_0(^1H)$ = 399.81 MHz, $\nu_0(^{13}C)$ = 100.54 MHz, and $\nu_0(^{15}N)$ = 40.52 MHz. Spectra were collected with a 4.0 mm Varian T3 double resonance (HX) magic angle-spinning (MAS) probe with samples packed into 4.0 mm zirconia rotors, or a 7.5 mm Varian T3 HX MAS probe with samples packed into 7.5 mm zirconia rotors. High-field ssNMR experiments were conducted at the National High Magnetic Field Laboratory (NHMFL; Tallahassee, FL) using a Bruker Avance NEO console and a home-built 21.1 T ultra-wide bore magnet,[1] with corresponding Larmor frequencies of $\nu_0(^1H)$ = 894.52 MHz and $\nu_0(^{35}Cl)$ = 87.64 MHz. Additional high-field experiments were conducted at 18.8 T using a Bruker Avance NEO console, with $\nu_0(^1H)$ = 800.14 MHz and $\nu_0(^{35}Cl)$ = 78.39 MHz. Home-built 5.0 mm HX low-E probes were used for all experiments with samples packed into cylindrical 5.0 mm o.d. sample containers designed at the NHMFL. All ssNMR spectra were processed and analyzed using the ssNake v1.4 software package.[2]

^{13}C Spectra. ^{13}C spectra were obtained using the ramped-amplitude ^{1}H-^{13}C CP/MAS pulse sequence.[3-6] Spinning rates of 10 to 12 kHz was employed. The ^{1}H-^{13}C CP/MAS pulse sequence used a 2.0 μs π/2 ^{1}H pulse and TPPM decoupling with a 9° phase modulation.[7] ^{1}H magnetization was transferred in 3.0 ms under Hartman–Hahn conditions.[8] A 50 ms acquisition length was used to digitize a 50 kHz wide spectrum. The signal was averaged for *ca*. 21 h and 33 h. The recycle delay was determined by the value of $T_1(^1H)$, which was found by the inversion recovery pulse sequence adapted for CP. Optimal recycle delays between 2 s and 15 s were used. The FID was Fourier transformed with 25 Hz of line broadening and zero filled to 8K points. The ^{13}C spectra were referenced externally to TMS by measuring the resonance frequency of the methyl carbon in 3-methyl glutaric acid at 18.84 ppm.[9]

^{15}N Spectra. ^{15}N spectra were obtained using the ramped amplitude ^{1}H-^{15}N CP/MAS pulse sequence applied to natural-abundance samples. A spinning rate of 5 kHz was employed. The ^{1}H-^{15}N CP/MAS pulse sequence used a 4.4 μs π/2 ^{1}H pulse and SPINAL-64 decoupling with a 6° phase modulation.[10] ^{1}H magnetization was transferred in 3.0 ms under Hartman–Hahn conditions. A 50 ms acquisition length was used to digitize a 50 kHz wide spectrum. The signal was averaged between *ca*. 36 h and 60 h for the fast MAS experiments, and between *ca*. 3 and 7 days for slaw

MAS experiments. The FID was Fourier transformed with 15 Hz of line broadening and zero filled. The ^{15}N spectra were referenced externally to a primary reference of nitromethane at 0.0 ppm by measuring the resonance frequency of glycine at 33.40 ppm.[11]

^{35}Cl Spectra. For **ERL$_2$·ZnCl$_2$**, direct-excitation experiments were performed under static conditions at 21.1 T using the WURST-CPMG pulse sequence.[12-15] Experiments used a recycle delay of 0.5 s and a total of 394,240 scans were collected (total experiment time of *ca*. 55 h). The spectra were acquired using 100 μs WURST-80 pulses with a sweep width of 1.5 MHz. A dephasing period of 150 μs was used. A continuous-wave decoupling field of $\nu_2(^1H)$ = 25 kHz was applied during all experiments. For **ERL·HCl**, direct-excitation experiments were performed under static conditions at 18.8 T using the CPMG pulse sequence.[16] Experiments used a recycle delay of 1.0 s and a total of 16,384 scans were collected (total experiment time of *ca*. 3.7 h). A continuous-wave decoupling field of $\nu_2(^1H)$ = 25 kHz was applied during all experiments. For both sets of data, echoes were co-added in the time domain prior to Fourier transformation and processed in magnitude mode. Uncertainties in the quadrupolar and chemical shift tensor parameters were assessed *via* bidirectional variation of each parameter, and visual comparison of experimental and simulated spectra. Chemical shifts were referenced externally to NaCl(s) at $\delta_{iso}(^{35}Cl) = -41.1$ ppm.

Supplement S2. Computational details of predicting NMR parameters

Geometry Optimizations. Structural refinements were performed using plane-wave DFT as implemented in the CASTEP module of BIOVIA Materials Studio 2020.[17] These calculations used as initial structural models the previously reported X-ray diffraction structures, or the structure of **$ERL_2{\cdot}ZnCl_2$** reported in this study. Calculations employed the PBE functional,[18] ultrasoft pseudopotentials generated *on-the-fly*,[19] a plane-wave cutoff energy of 800 eV, and a Monkhorst-Pack grid with a *k*-point spacing of 0.05 $Å^{-1}$.[20] Semiempirical dispersion corrections were introduced through the many-body dispersion model of Tkatchenko.[21] Structural refinements employed the LBFGS energy-minimizing scheme,[22] in which the positions of all atoms were relaxed while holding unit cell parameters constant. Convergence thresholds included a maximum change in energy of 5×10^{-6} eV $atom^{-1}$, a maximum displacement of 5×10^{-4} Å $atom^{-1}$, and a maximum Cartesian force of 10^{-2} eV $Å^{-1}$.

Magnetic Shielding and Electric Field Gradient Tensors. Magnetic shielding tensors were calculated in CASTEP using the gauge-including projector-augmented wave (GIPAW) method[23] and in AMS using the gauge-including atomic orbital (GIAO) method.[24, 25] CASTEP calculations used structural models consisting of the fully periodic crystal structure or an isolated molecule, whereas AMS calculations were performed on isolated molecules only. CASTEP calculations of EFG and magnetic shielding tensors used the PBE functional, ultrasoft pseudopotentials generated *on-the-fly*, a plane-wave cutoff energy of 800 eV, and a Monkhorst-Pack grid with a *k*-point spacing of 0.05 $Å^{-1}$. AMS calculations of magnetic shielding tensors used the TZ2P basis set (or TZ2P+ for zinc atoms), along with the hybrid functional PBE0,[26] which is known to perform well for the prediction of NMR interaction tensors in a variety of materials.

^{13}C and ^{15}N magnetic shielding tensors and ^{35}Cl EFG tensors were obtained from three separate calculations: (i) a PBE calculation in CASTEP using the periodic crystal lattice as a structural model; (ii) a PBE calculation in CASTEP using only an isolated molecule, which was approximated using a *P*1 unit cell with a size of 35 × 35 × 35 Å; and (iii) a final PBE0 calculation in AMS using an isolated molecule as the structural model. The difference between the first two calculations provides the intermolecular contributions to the ^{13}C and ^{15}N magnetic shielding tensors and ^{35}Cl EFG tensors, which are added to the higher level PBE0 calculation performed in AMS. The advantage of this method rests on the fact that magnetic shielding is

largely a local phenomenon, meaning that the influences of weak noncovalent interactions on magnetic shielding tensors can be calculated at a tractable lower level.[27-31]

Calculated ^{13}C and ^{15}N magnetic shielding tensors were converted to the chemical shift scale through additional calculations of a series of systems (L-histidine HCl H_2O, L-asparagine H_2O, imidazole, cytosine, and uracil) that have been characterized in previous ssNMR studies using least-squares linear regression analysis (**Figure S1**).[30, 32] Calculated ^{35}Cl magnetic shieldings were converted to the chemical shift scale by setting the computed shielding for L-histidine HCl H_2O to 34.5 ppm.[33]

Comparison between Calculation and Experiment. The relationship between calculated principal components of the magnetic shielding tensors ($\sigma_{ii}^{v,\mathrm{calc}}$) and experimental principal components of chemical shift tensors ($\delta_{ii}^{v,\mathrm{exp}}$) is described by the following expression:

$$\sigma_{ii}^{v,\mathrm{calc}} = A\delta_{ii}^{v,\mathrm{exp}} + B. \qquad \textbf{Eq. S1}$$

where the index v denotes the carbon or nitrogen site (v = 1, 2, …, N), the index i denotes the principal component of the shielding tensor (i = 1, 2, 3), A represents the slope of the correlation line, and B represents the interpolated shielding of the reference state (*i.e.*, tetramethylsilane at $\delta_{\mathrm{iso}}(^{13}C)$ = 0.0 ppm; nitromethane at $\delta_{\mathrm{iso}}(^{15}N)$ = 0.0 ppm). Calculated chemical shifts ($\delta_{ii}^{v,\mathrm{calc}}$) are derived from the following expression:

$$\delta_{ii}^{v,\mathrm{calc}} = (B - \sigma_{ii}^{v,\mathrm{calc}})/A. \qquad \textbf{Eq. S2}$$

The *chemical shift distance* for atom *v, d_v*, provides a comparison between a calculated and experimental chemical shift tensor with a single scalar value in ppm. Given two sets of principal components of chemical shift tensors, d_v is defined by the following expression:[34]

$$d_v = \left(\frac{1}{15}\Big[3\big(\delta_{11}^{v,\mathrm{calc}} - \delta_{11}^{v,\mathrm{exp}}\big)^2 + 3\big(\delta_{22}^{v,\mathrm{calc}} - \delta_{22}^{v,\mathrm{exp}}\big)^2 + 3\big(\delta_{33}^{v,\mathrm{calc}} - \delta_{33}^{v,\mathrm{exp}}\big)^2 + 2\big(\delta_{11}^{v,\mathrm{calc}} - \delta_{11}^{v,\mathrm{exp}}\big)\big(\delta_{22}^{v,\mathrm{calc}} - \delta_{22}^{v,\mathrm{exp}}\big) + 2\big(\delta_{11}^{v,\mathrm{calc}} - \delta_{11}^{v,\mathrm{exp}}\big)\big(\delta_{33}^{v,\mathrm{calc}} - \delta_{33}^{v,\mathrm{exp}}\big) + 2\big(\delta_{22}^{v,\mathrm{calc}} - \delta_{22}^{v,\mathrm{exp}}\big)\big(\delta_{33}^{v,\mathrm{calc}} - \delta_{33}^{\mathrm{exp}}\big)\Big]\right)^{1/2}. \qquad \textbf{Eq. S3}$$

A root-mean-square chemical shift distance for N chemical shift tensors (Δ_{RMS}) is determined by the following expression:

$$\Delta_{\mathrm{RMS}} = \left(\frac{1}{N} \sum_{v=1}^{N} d_v^2 \right)^{1/2} . \qquad \textbf{Eq. S4}$$

The value for the shielding of the reference system, B, and the slope, A, are optimized numerically for each type of calculation to minimize the value of Δ_{RMS}.

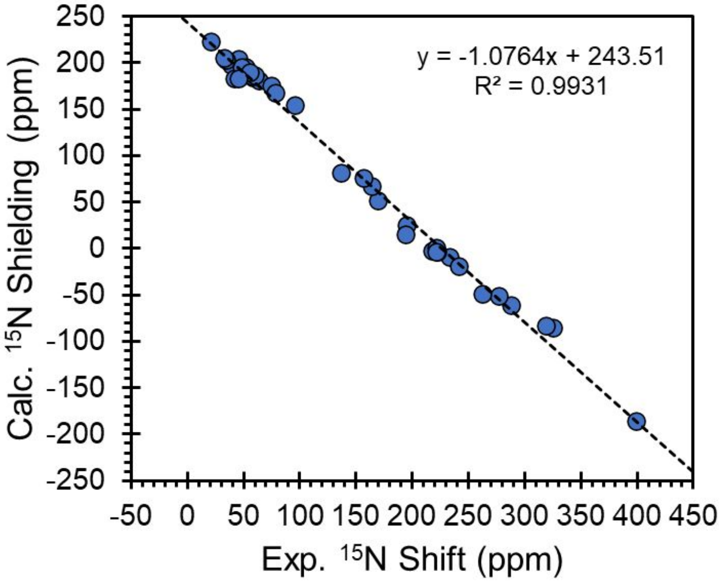

Figure S1. Correlation between the principal values of experimental ^{15}N chemical shift tensors and principal components of calculated ^{15}N magnetic shielding tensors. Results are shown for L-histidine HCl H_2O, L-asparagine H_2O, imidazole, cytosine, and uracil.

Supplement S3. Differential Scanning Calorimetry

DSC was performed on a TA Instruments Q20 DSC calibrated with an indium reference standard. Approximately 5-10 mg of sample was crimp sealed in an aluminum sample pan. The samples were heated from 50 ºC to 300 ºC with a ramp rate of 10 ºC min^{-1} under nitrogen purging atmosphere with a 50 mL min^{-1} flow rate. TA Universal Analysis software was used to evaluate the data.

The DSC (**Figure S2**) trace for **ERL·HCl** exhibits a phase transition at 236 °C, consistent with melting, followed by a second transition at 265 °C, consistent with decomposition.[35] **ERL·H_2O** exhibits two transitions between 60 °C and 100 °C that are associated with water loss, as well as a transition from melting or decomposition at 165 °C.[36] The difference in melting points between **ERL·HCl** and **ERL·H_2O** is consistent with the common observation that salts have higher thermal stability. **ERL_2·$ZnCl_2$** shows a small endotherm at approximately 234 °C, immediately followed by an exothermic event and shifted baseline, consistent with volatilization and/or weight loss following the melting transition.

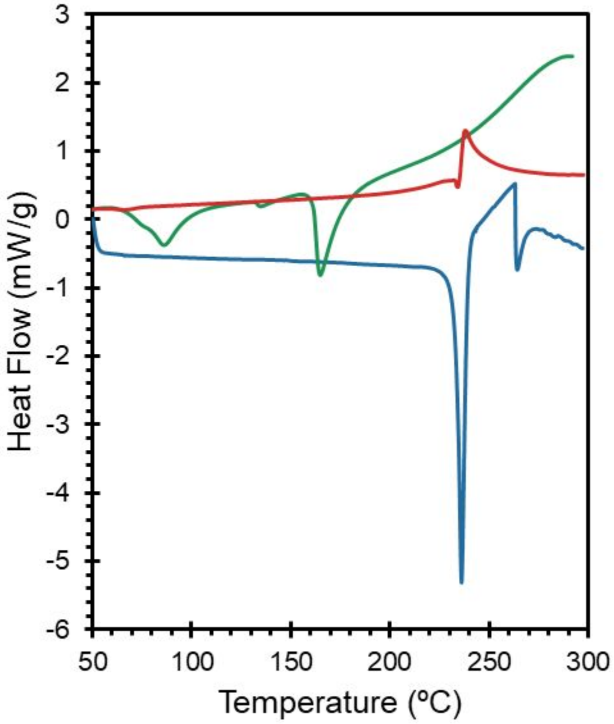

Figure S2. Differential scanning calorimetry thermograms for three solid forms of erlotinib. The materials are colored as follows: **ERL·HCl** in blue, **ERL·H_2O** in green, and **ERL_2·$ZnCl_2$** in red.

Supplement S4. X-ray Photoelectron Spectroscopy

XPS measurements were performed using a Physical Electronics PHI 5000 VersaProbe system equipped with a monochromatic Al *K*α X-ray source at 1486.6 eV with a 100 μm beam size (**Figure S3**). Measurements were carried out at room temperature at a pressure below 10^{-8} torr. Compositional survey scans were obtained using a pass energy of 117.4 eV and energy step of 0.5 eV. High-resolution detailed scans of each element were acquired using a pass energy of 23.5 eV and energy step of 0.1 eV.

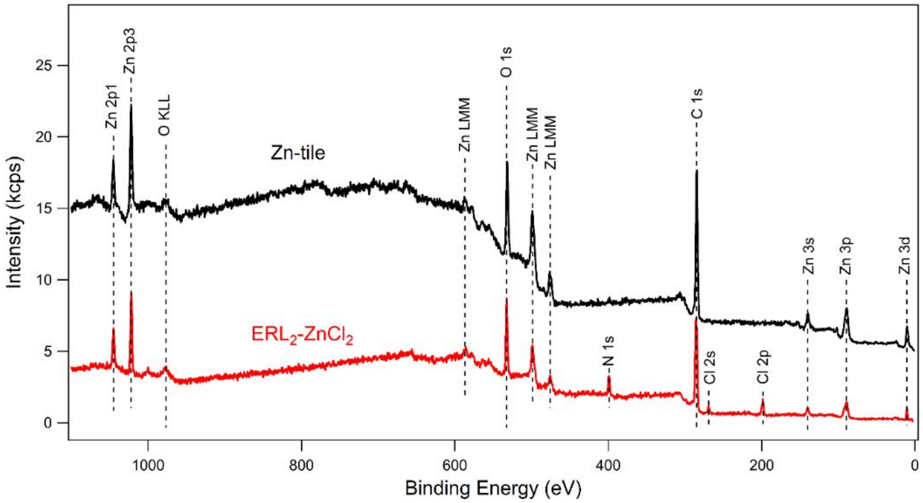

Figure S3. XPS compositional survey scans of **$ERL_2 \cdot ZnCl_2$** and the zinc tile used in its synthesis. In the Zn tile sample, only zinc, carbon, and oxygen were detected. The strong carbon signal in the Zn tile is attributed to adventitious carbon contamination, while the pronounced oxygen signal is a result of surface oxidation.

Supplement S5. Infrared Spectroscopy

ATR-IR was performed using a Thermo Scientific Nicolet iS50 FTIR. The scans were collected at a range of 500 cm^{-1} to 4000 cm^{-1}, with 256 scans at a resolution of 2 cm^{-1}. Samples were analyzed on the ATR stage and the empty stage was used as the background.

The peaks in the infrared spectra (**Figure S4, Table S1**) for each form of erlotinib at *ca*. 1000 cm^{-1} corresponds to the aromatic ring, at *ca*. 1250 cm^{-1} to C-O stretching, at *ca*. 1590 cm^{-1} to C-H, O-H or N-H single bond bending, and at *ca*. 2110 cm^{-1} to the alkyne. **ERL·H_2O** features a broad peak at 3486 cm^{-1} due to the presence of water molecules.[37] **ERL·H_2O** and **ERL_2·$ZnCl_2$** have additional bands at *ca*. 1470 cm^{-1}, likely from C-H single bond bending. **ERL_2·$ZnCl_2$** has extra peaks around 1500 cm^{-1} that are larger and sharper than those observed for the other forms, possibly from the same single bond bending or from C=C double bond stretching. There are two peaks for **ERL_2·$ZnCl_2$** at 1516 cm^{-1} and 1434 cm^{-1}, indicating that there is no center of inversion in either molecule according to the rule of mutual exclusion.[37]

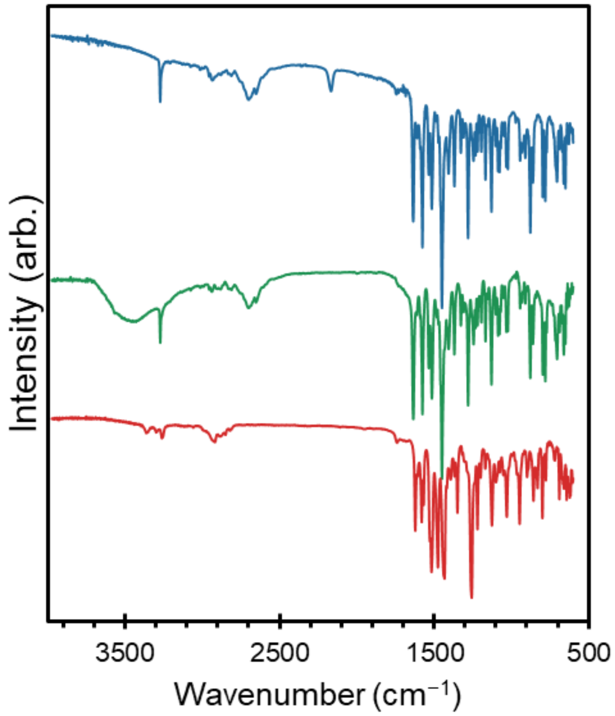

Figure S4. Attenuated total reflectance infrared spectra for three solid forms of erlotinib. The materials are colored as follows: **ERL·HCl** in blue, **ERL·H_2O** in green, and **ERL_2·$ZnCl_2$** in red.

Table S1. Summary of peak positions of the ATR-IR of three forms of erlotinib.

Spectral Range (cm^{-1})	ERL·HCl	ERL·H_2O	ERL_2·$ZnCl_2$
3200 – 3600	One peak 3272	One peak 3486	One peak 3261
2800 – 3200	-	-	One peak 2918
2400 – 2800	-	-	-
2000 – 2400	One peak 2166	-	-
1600 – 2000	One peak 1635	One peak 1631	One peak 1621
1400 – 1600	Three sharp peaks 1575, 1514, 1448	Four peaks 1580, 1512, 1468 and 1435	Four peaks 1580, 1516, 1475, and 1432
1200 – 1400	Two peaks 1368 and 1280	Two peaks 1248 and 1212	Three peaks 1348, 1256 and 1219
1000 – 1200	One peak 1128	-	Two peaks 1125 and 1029
800 – 1000	One peak 877	One peak 872	One peak 946
600 – 800	Two peaks 797 and 780	Three peaks 787, 687, and 662	One peak 799

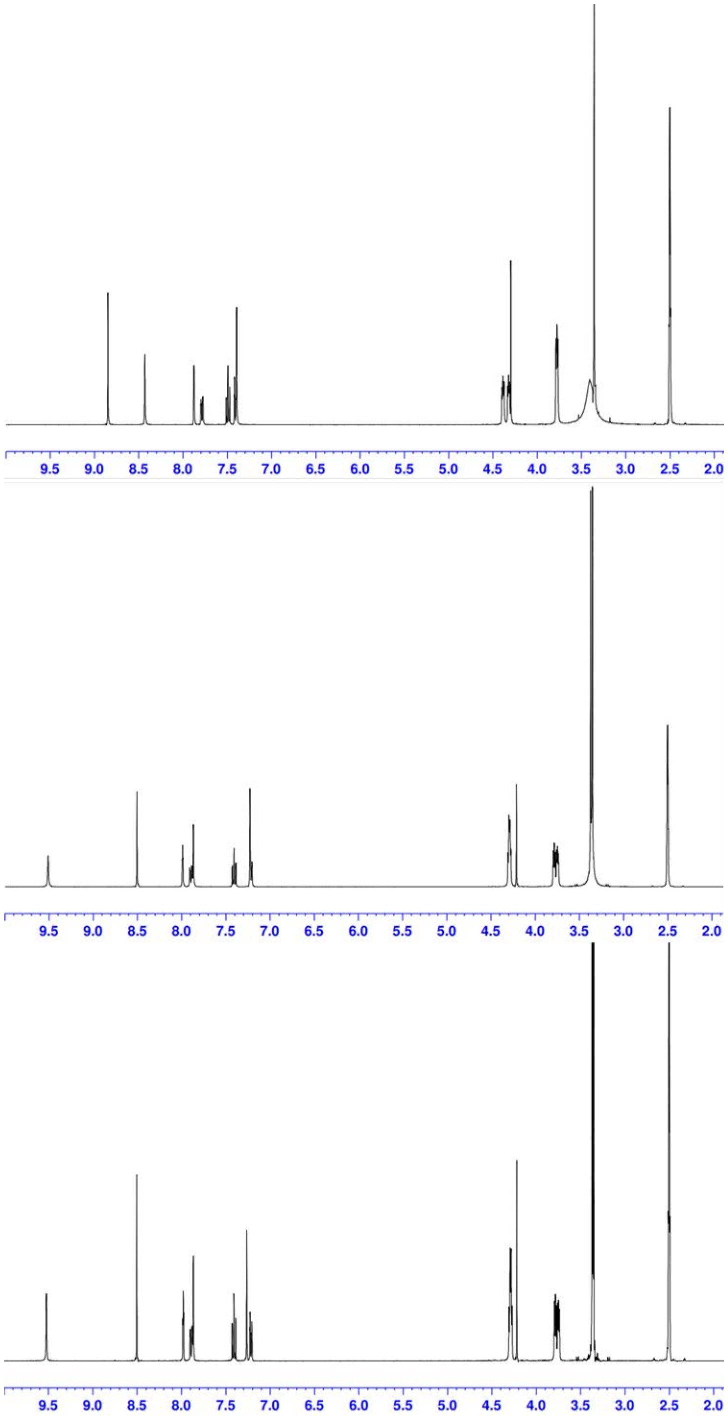

Figure S5a. Solution-state ^{1}H NMR spectra of **ERL·HCl** (top), **ERL·H_2O** (middle), and **ERL_2·$ZnCl_2$** (bottom) show that the proton shifts of the zinc complex match the freebase. This indicates that the complex dissociates into the freebase when dissolved in DMSO solvent. NMR spectra were recorded on a Bruker 400 MHz Ascend Spectrometer at 21 °C. ^{1}H chemical shifts were referenced with respect to TMS (0.0 ppm) using DMSO-d_6 as a secondary standard (39.51 ppm).

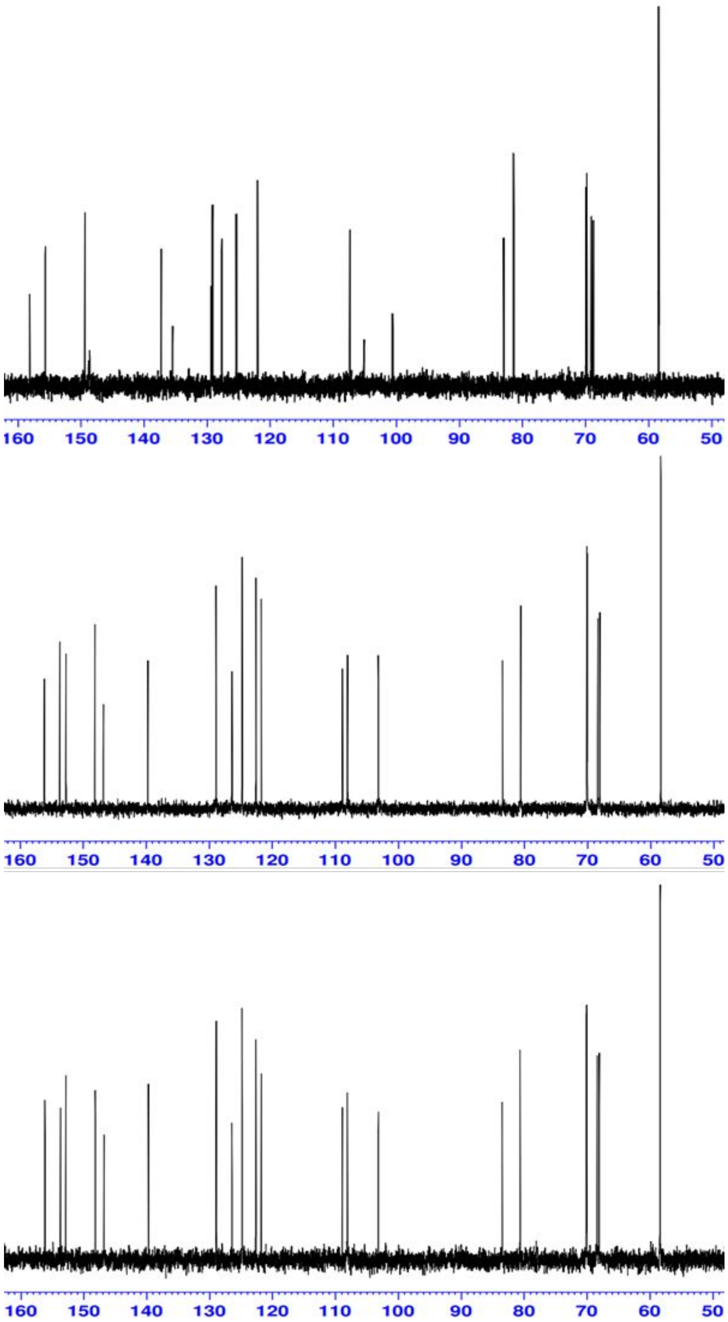

Figure S5b. Solution-state ^{13}C NMR spectra of **ERL·HCl** (top), **ERL·H$_2$O** (middle), and **ERL$_2$·ZnCl$_2$** (bottom) show that the carbon shifts of the zinc complex match the freebase. This indicates that the complex dissociates into the freebase when dissolved in DMSO solvent. NMR spectra were recorded on a Bruker 400 MHz Ascend Spectrometer at 21 °C. ^1H chemical shifts were referenced with respect to TMS (0.0 ppm) using DMSO-d_6 as a secondary standard (39.51 ppm).

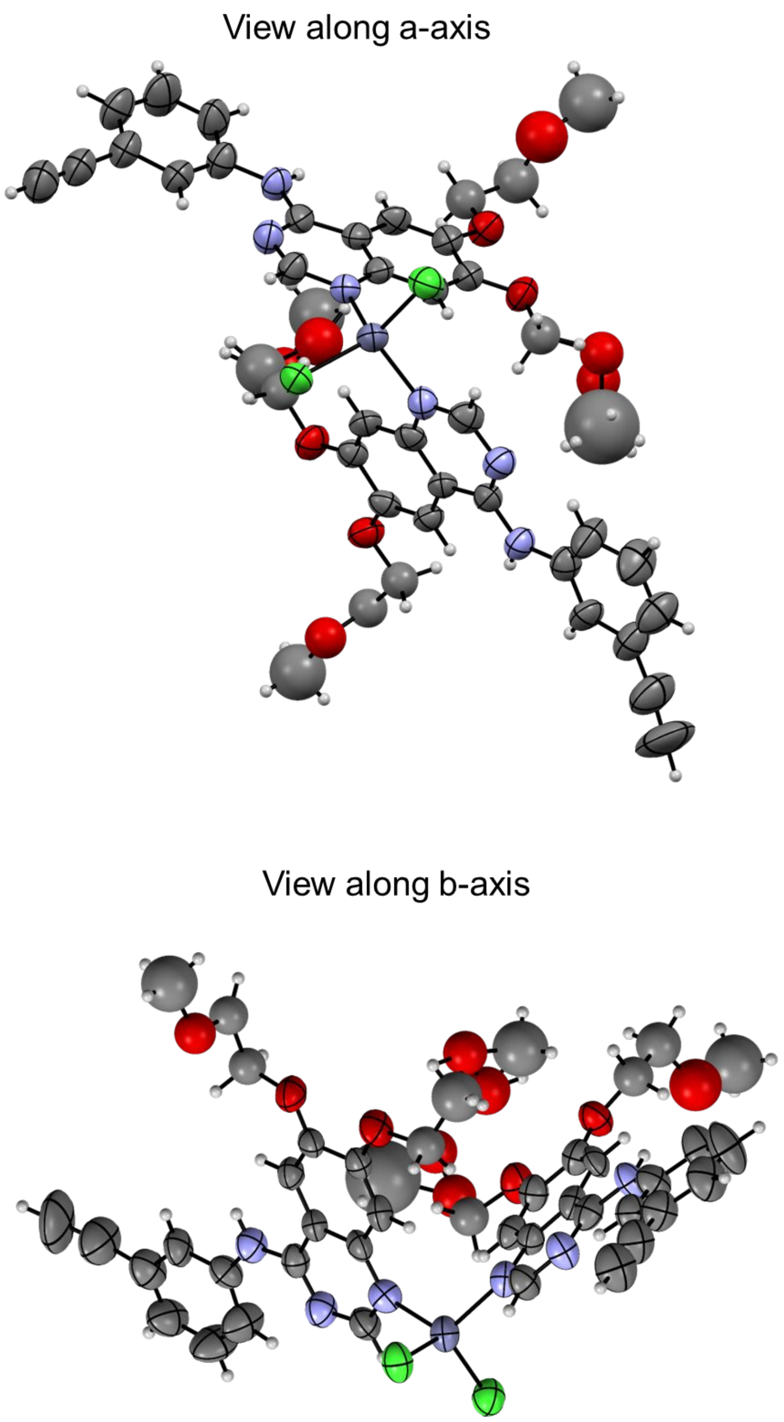

Figure S6. ORTEP images of the crystal structure of **ERL$_2$·ZnCl$_2$** viewed along the crystallographic *a*- and *b*-axes, as determined by single-crystal X-ray diffraction.

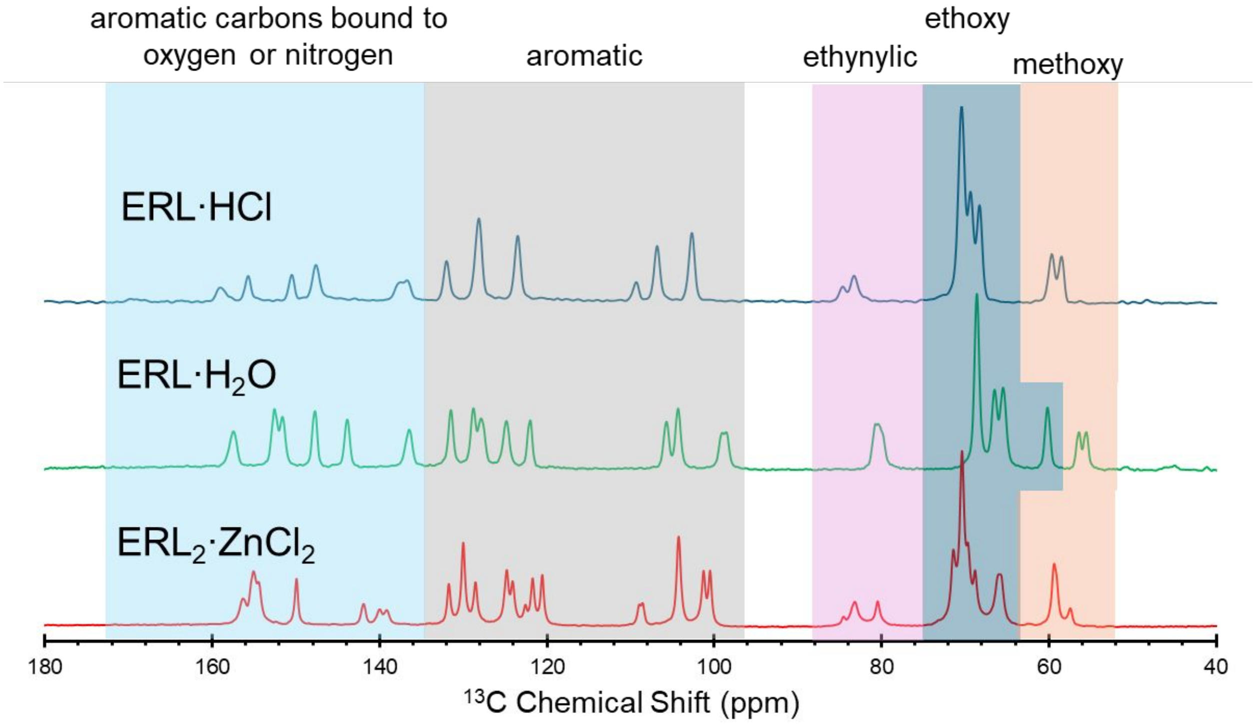

Figure S7. ^{1}H-^{13}C CP/MAS spectra of three forms of erlotinib, along with approximate assignments of different chemical moieties provided by DFT calculations.

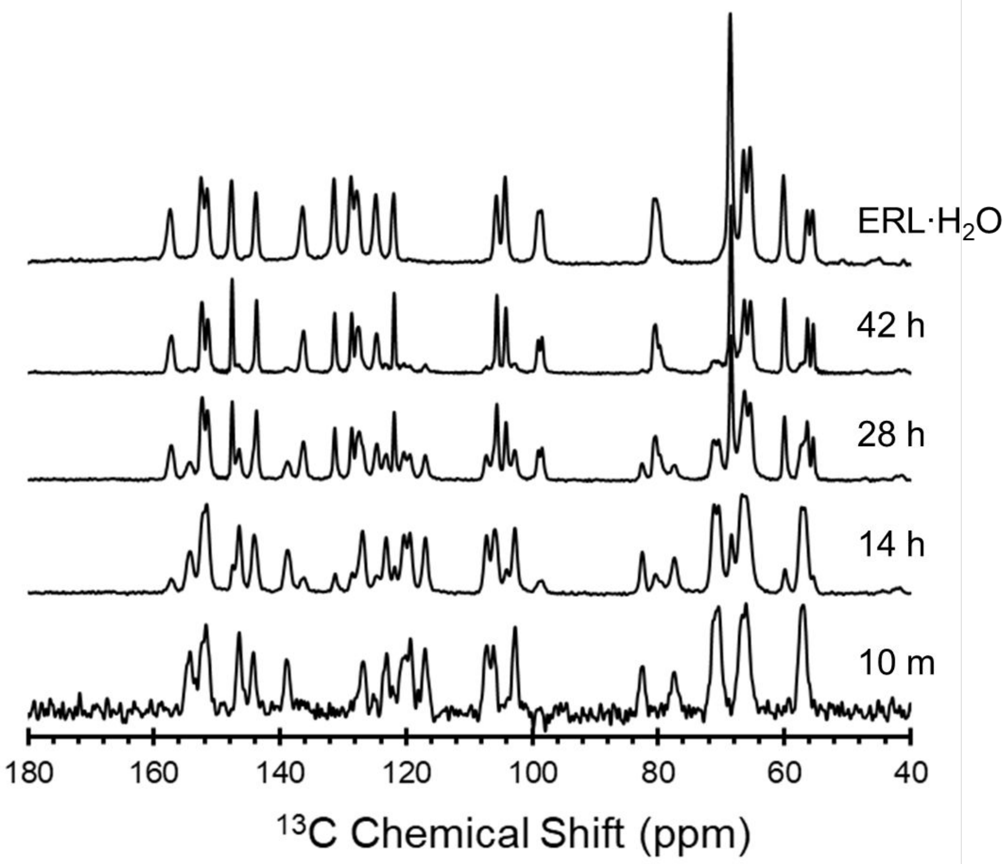

Figure S8. ^{1}H-^{13}C CP/MAS spectra of **ERL·FB** collected over a period of 42 h. The spectra reveal the gradual conversion to **ERL·H_2O**. A spectrum of **ERL·H_2O** is shown for comparison.

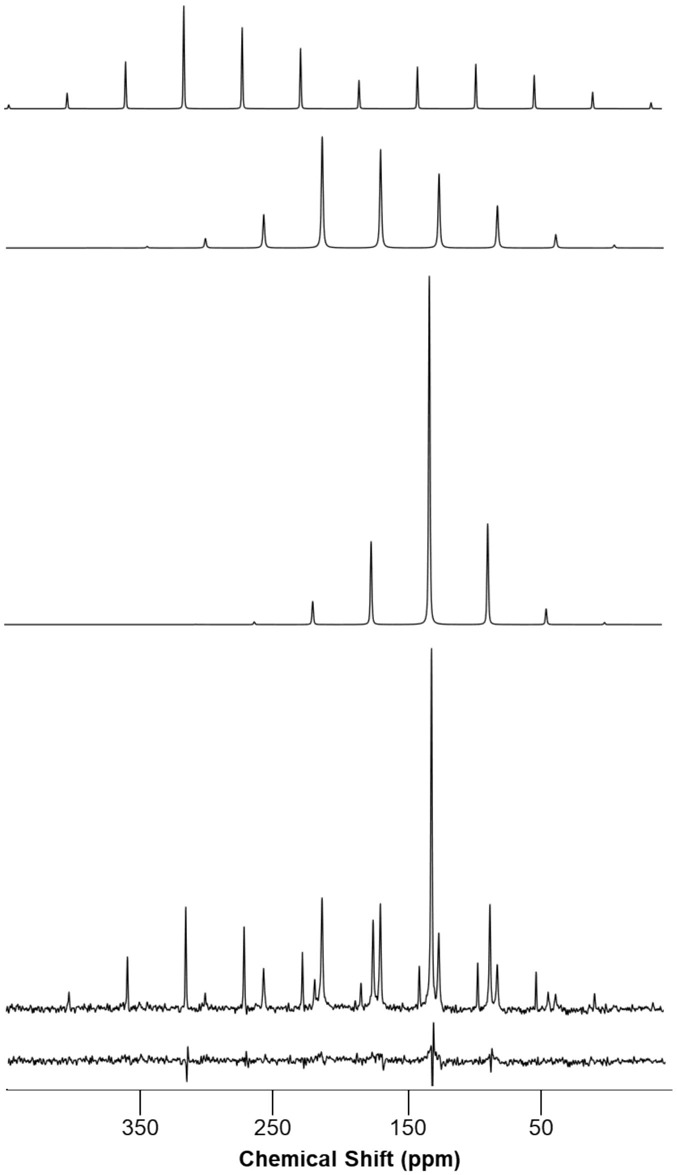

Figure S9a. The $^{1}H \rightarrow ^{15}N$ CP/MAS spectrum of **ERL·HCl** with a spin rate of 1800 Hz is shown. The intensity of the spinning sidebands reflects the anisotropy. The simulations of peaks associated with N^2 (top), N^1 (middle), and N^3 (bottom) are stacked by isotropic shift.

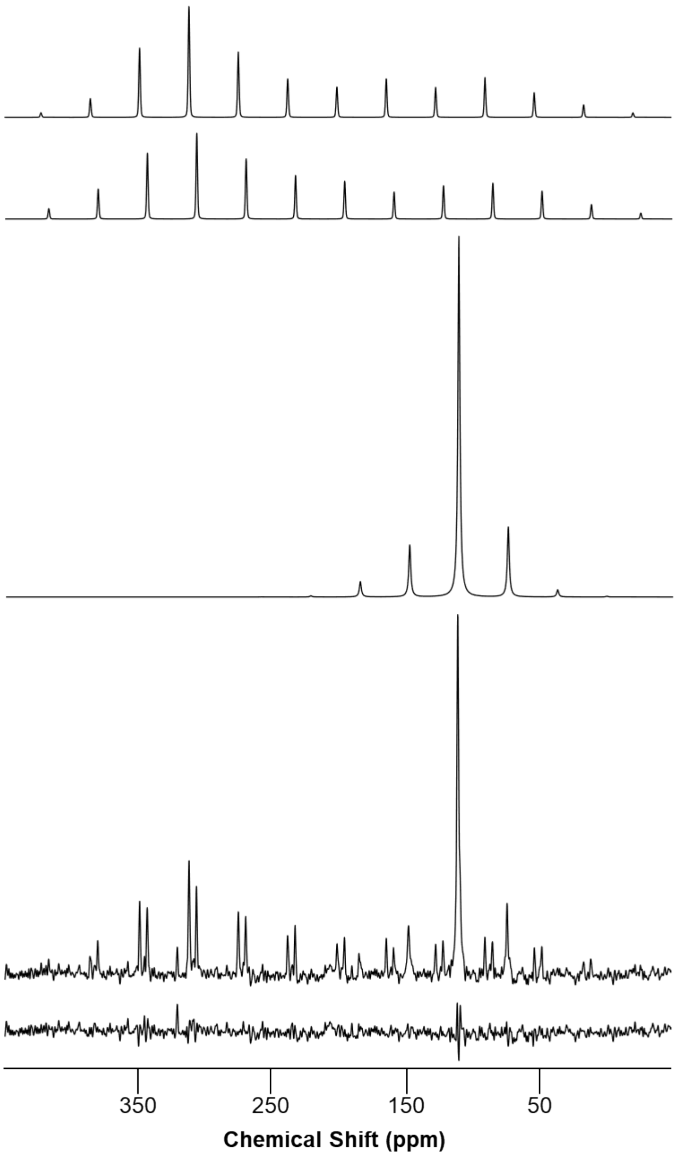

Figure S9b. The $^{1}H \rightarrow ^{15}N$ CP slow MAS spectrum of **ERL·H_2O** with a spin rate of 1500 Hz is shown. The intensity of the spinning sidebands reflects the anisotropy. The simulations of peaks associated with N^1 (top), N^2 (middle), and N^3 (bottom) are stacked by isotropic shift.

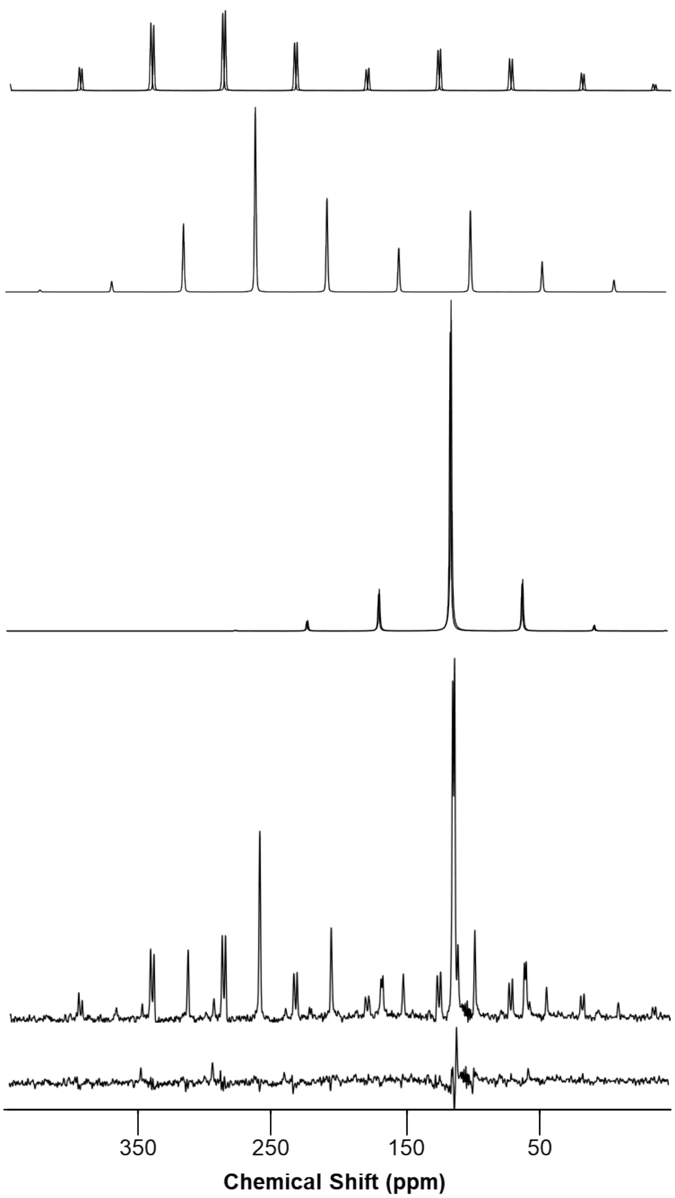

Figure S9c. The $^1H \rightarrow ^{15}N$ CP slow MAS spectrum of **$ERL_2 \cdot ZnCl_2$** with a spin rate of 2200 Hz is shown. The intensity of the spinning sidebands reflects the anisotropy. The simulations of peaks associated with N^2 (top), N^1 (middle), and N^3 (bottom) are stacked by isotropic shift.

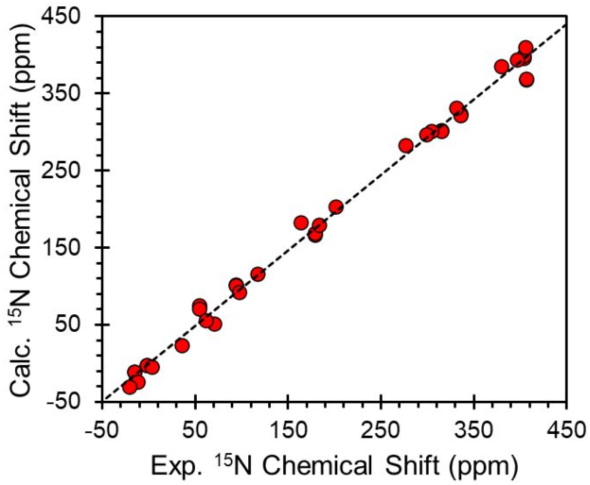

Figure S10. Correlation between calculated and experimental ^{15}N chemical shift tensor principal components for **ERL·H_2O** and **ERL_2·$ZnCl_2$**.

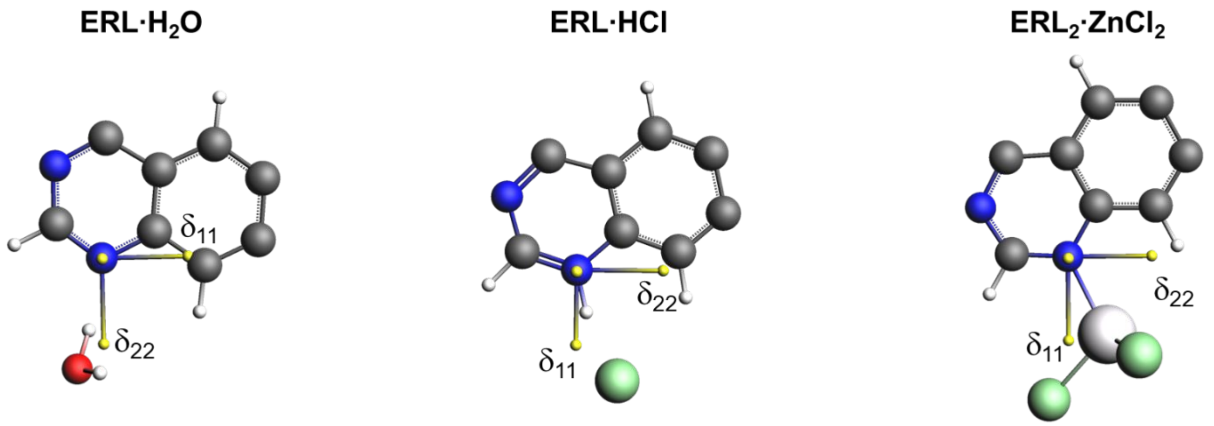

Figure S11. Orientations of the ^{15}N chemical shift tensor of N^1 for three solid forms of erlotinib. In all cases, the orientation of δ_{33} is perpendicular to the page.

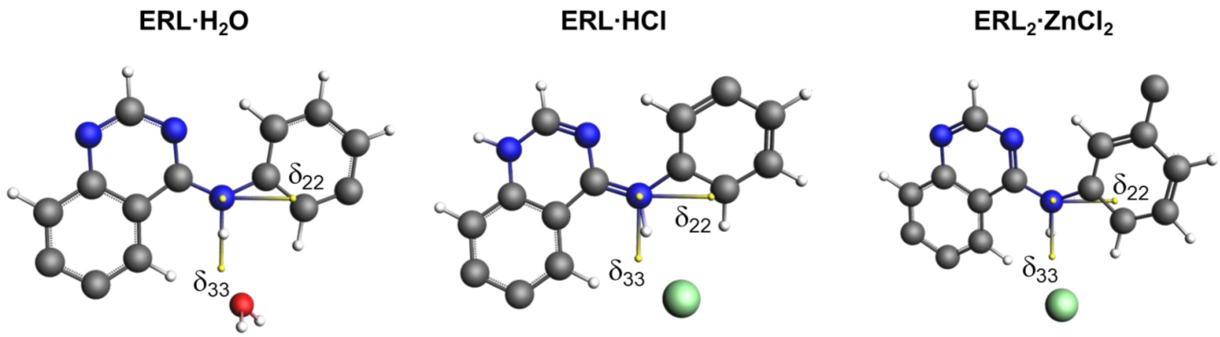

Figure S12. Orientations of the ^{15}N chemical shift tensor of N^3 for three solid forms of erlotinib. In all cases, the orientation of δ_{11} is perpendicular to the page.

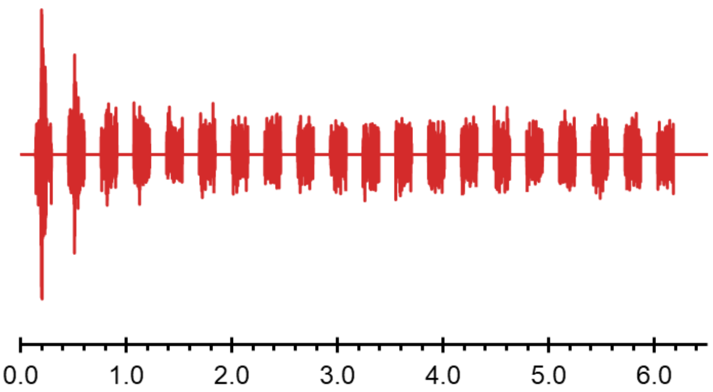

Figure S13. Time domain ^{35}Cl ssNMR spectrum of **ERL$_2$·ZnCl$_2$** acquired at 21.1 T using the WURST-CPMG pulse sequence, illustrating the low value of T_2^{eff}.

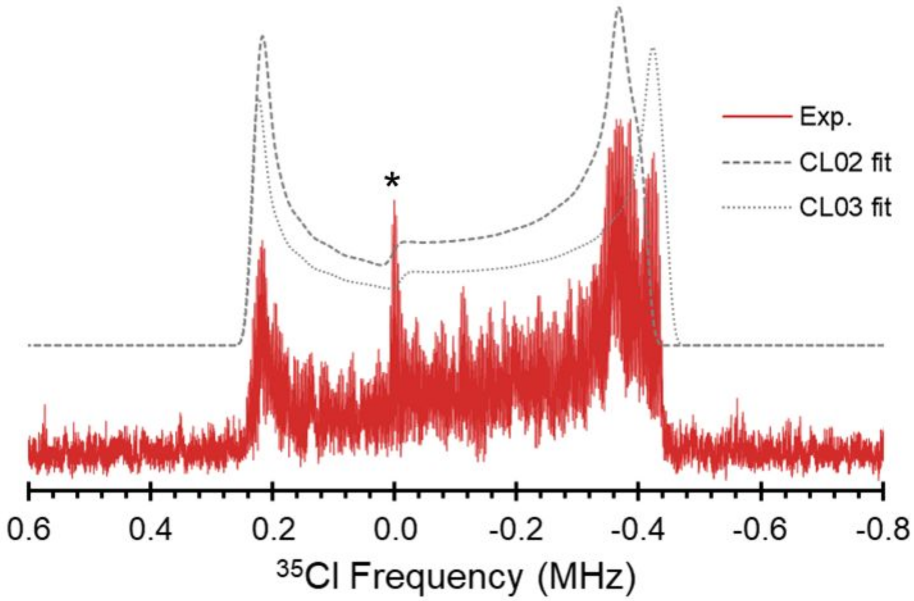

Figure S14. A ^{35}Cl ssNMR spectrum of **ERL$_2$·ZnCl$_2$** acquired at 21.1 T using the WURST-CPMG pulse sequence (red), along with a simulated pattern (black) and a deconvolution of the pattern into the two underlying components, corresponding to the two types of chlorine atoms (CL02 and CL03). The asterisk indicates an additional signal that may correspond to a small amount of **ERL·HCl**.

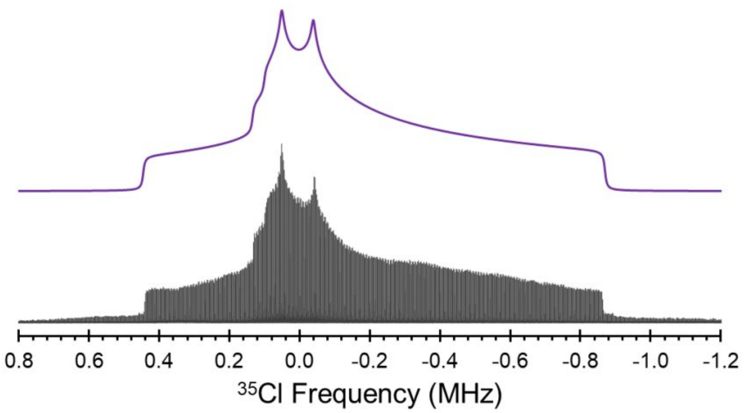

Figure S15. Experimental ^{35}Cl ssNMR spectrum of $ZnCl_2$ obtained at 21.1 T in four pieces (gray) and corresponding analytical simulation (purple).

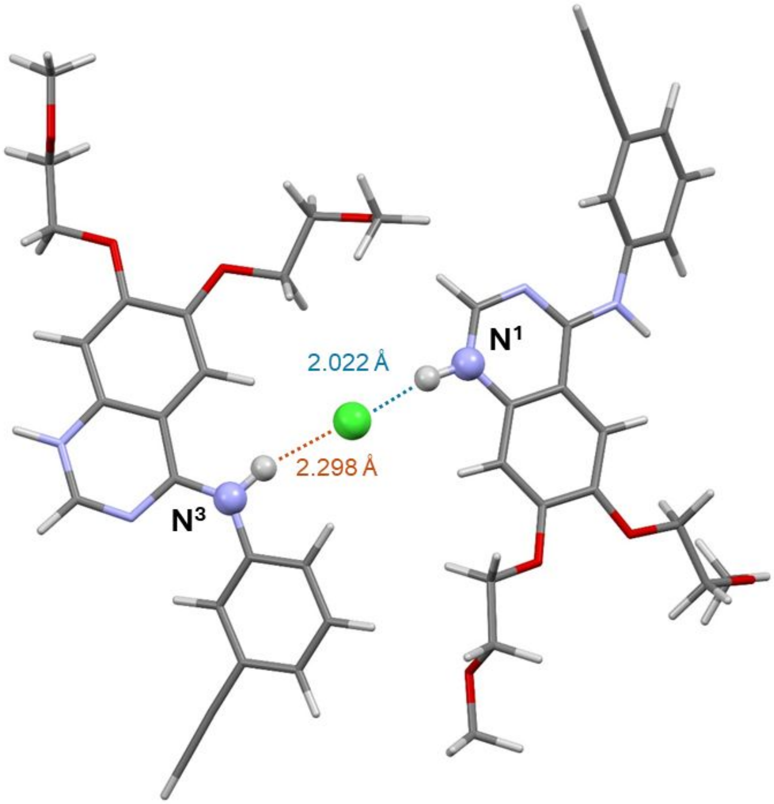

Figure S16. Illustration of the H···Cl^- hydrogen bonds in a polymorph of **ERL·HCl** (CSD code MIYBOM). The distances indicated are derived from the geometry-optimized structure. The nitrogen atoms N^1 and N^3 are indicated.

Table S2. Summary of experimental ^{35}Cl EFG and chemical shift tensor parameters for ERL-HCl. [a-d]

C_Q (MHz)	η_Q	δ_{iso} (ppm)	Ω (ppm)	κ	α (°)	β (°)	γ (°)
7.48(6)	0.13(3)	74(5)	100(50)	< 0	80(10)	10(30)	0(30)

[a] The experimental uncertainties in the last digit for each value are indicated in parentheses.

[b] The principal components of the EFG tensors are defined such that $|V_{33}| \geq |V_{22}| \geq |V_{11}|$. The quadrupolar coupling constant and asymmetry parameter are given by $C_Q = eQV_{33}/h$, and $\eta_Q = (V_{11} - V_{22})/V_{33}$, respectively. The sign of C_Q cannot be determined from the experimental ^{35}Cl spectra.

[c] The chemical shift tensors are defined such that the principal components are ordered $\delta_{11} \geq \delta_{22} \geq \delta_{33}$. The isotropic chemical shift, span, and skew parameter are given by $\delta_{iso} = \delta_{11} - \delta_{33}$, $\kappa = 3(\delta_{22} - \delta_{iso})/\Omega$, respectively.

[d] The Euler angles α, β, and γ define the relative orientation of the EFG and chemical shift tensors using the ZY′Z″ convention for rotation.

References

(1) Fu, R.; Brey, W. W.; Shetty, K.; Gor'kov, P.; Saha, S.; Long, J. R.; Grant, S. C.; Chekmenev, E. Y.; Hu, J.; Gan, Z.; et al. Ultra-wide bore 900 MHz high-resolution NMR at the National High Magnetic Field Laboratory. *J. Magn. Reson.* **2005**, *177*, 1-8.
(2) van Meerten, S. G. J.; Franssen, W. M. J.; Kentgens, A. P. M. ssNake: a cross-platform open-source NMR data processing and fitting application. *J. Magn. Reson.* **2019**, *301*, 56-66.
(3) Peersen, O. B.; Wu, X. L.; Kustanovich, I.; Smith, S. O. Variable-amplitude cross-polarization MAS NMR. *J. Magn. Reson.* **1993**, *104*, 334-339.
(4) Metz, G.; Wu, X. L.; Smith, S. O. Ramped-amplitude cross polarization in magic-angle-spinning NMR. *J. Magn. Reson.* **1994**, *110*, 219-227.
(5) Schaefer, J.; Stejskal, E. O. Carbon-13 nuclear magnetic resonance of polymers spinning at the magic angle. *J. Am. Chem. Soc.* **1976**, *98*, 1031-1032.
(6) Pines, A.; Gibby, M. G.; Waugh, J. S. Proton-enhanced nuclear induction spectroscopy ^{13}C chemical shielding anisotropy in some organic solids. *Chem. Phys. Lett.* **1972**, *15*, 373-376.
(7) Bennett, A. E.; Rienstra, C. M.; Auger, M.; Lakshmi, K. V.; Griffin, R. G. Heteronuclear decoupling in rotating solids. *J. Chem. Phys.* **1995**, *103*, 6951-6958.
(8) Hartmann, S. R.; Hahn, E. L. Nuclear double resonance in the rotating frame. *Phys. Rev.* **1962**, *128*, 2042-2053.
(9) Barich, D. H.; Gorman, E. M.; Zell, M. T.; Munson, E. J. 3-methylglutaric acid as a ^{13}C solid-state NMR standard. *Solid State Nucl. Magn. Reson.* **2006**, *30*, 125-129.
(10) Fung, B. M.; Khitrin, A. K.; Ermolaev, K. An improved broadband decoupling sequence for liquid crystals and solids. *J. Magn. Reson.* **2000**, *142*, 97-101.
(11) Bertani, P.; Raya, J.; Bechinger, B. ^{15}N chemical shift referencing in solid state NMR. *Solid State Nucl. Magn. Reson.* **2014**, *61-62*, 15-18.
(12) O'Dell, L. A.; Rossini, A. J.; Schurko, R. W. Acquisition of ultra-wideline NMR spectra from quadrupolar nuclei by frequency stepped WURST–QCPMG. *Chem. Phys. Lett.* **2009**, *468*, 330-335.
(13) O'Dell, L. A.; Schurko, R. W. QCPMG using adiabatic pulses for faster acquisition of ultra-wideline NMR spectra. *Chem. Phys. Lett.* **2008**, *464*, 97-102.
(14) Bhattacharyya, R.; Frydman, L. Quadrupolar nuclear magnetic resonance spectroscopy in solids using frequency-swept echoing pulses. *J. Chem. Phys.* **2007**, *127*, 194503.
(15) Kupce, E.; Freeman, R. Adiabatic pulses for wideband inversion and broadband decoupling. *J. Magn. Reson.* **1995**, *115*, 273-276.
(16) Lipton, A. S.; Sears, J. A.; Ellis, P. D. A general strategy for the NMR observation of half-integer quadrupolar nuclei in dilute environments. *J. Magn. Reson.* **2001**, *151*, 48-59.
(17) Clark, S. J.; Segall, M. D.; Pickard, C. J.; Hasnip, P. J.; Probert, M. J.; Refson, K.; Payne, M. C. First principles methods using CASTEP. *Z. Kristallogr.* **2005**, *220*, 567-570.
(18) Perdew, J. P.; Burke, K.; Ernzerhof, M. Generalized gradient approximation made simple. *Phys. Rev. Lett.* **1996**, *77*, 3865-3868.
(19) Yates, J. R.; Pickard, C. J.; Mauri, F. Calculation of NMR chemical shifts for extended systems using ultrasoft pseudopotentials. *Phys. Rev. B* **2007**, *76*, 024401.
(20) Monkhorst, H. J.; Pack, J. D. Special points for Brillouin-zone integrations. *Phys. Rev. B* **1976**, *13*, 5188-5192.
(21) Tkatchenko, A.; DiStasio, R. A.; Car, R.; Scheffler, M. Accurate and efficient method for many-body van der Waals interactions. *Phys. Rev. Lett.* **2012**, *108*, 236402.
(22) Pfrommer, B. G.; Côté, M.; Louie, S. G.; Cohen, M. L. Relaxation of crystals with the quasi-Newton method. *J. Comput. Phys.* **1997**, *131*, 233-240.
(23) Pickard, C. J.; Mauri, F. All-electron magnetic response with pseudopotentials: NMR chemical shifts. *Phys. Rev. B* **2001**, *63*, 245101.
(24) Ditchfield, R. Self-consistent perturbation theory of diamagnetism. *Mol. Phys.* **1974**, *27*, 789-807.
(25) Wolinski, K.; Hinton, J. F.; Pulay, P. Efficient implementation of the gauge-independent atomic orbital method for NMR chemical shift calculations. *J. Am. Chem. Soc.* **1990**, *112*, 8251-8260.
(26) Adamo, C.; Barone, V. Toward reliable density functional methods without adjustable parameters: The PBE0 model. *J. Chem. Phys.* **1999**, *110*, 6158-6170.
(27) Dračínský, M.; Unzueta, P.; Beran, G. J. O. Improving the accuracy of solid-state nuclear magnetic resonance chemical shift prediction with a simple molecular correction. *Phys. Chem. Chem. Phys.* **2019**, *21*, 14992-15000.

(28) Hartman, J. D.; Harper, J. K. Improving the accuracy of GIPAW chemical shielding calculations with cluster and fragment corrections. *Solid State Nucl. Magn. Reson.* **2022**, *122*, 101832.
(29) Nakajima, T. An extrapolation scheme for solid-state NMR chemical shift calculations. *Chem. Phys. Lett.* **2017**, *677*, 99-106.
(30) Holmes, S. T.; Boley, C. M.; Dewicki, A.; Gardner, Z. T.; Vojvodin, C. S.; Iuliucci, R. J.; Schurko, R. W. Carbon-13 chemical shift tensor measurements for nitrogen-dense compounds. *Magn. Reson. Chem.* **2024**, *62*, 179-189.
(31) Iuliucci, R. J.; Hartman, J. D.; Beran, G. J. O. Do models beyond hybrid density functionals increase the agreement with experiment for predicted NMR chemical shifts or electric field gradient tensors in organic solids? *J. Phys. Chem. A* **2023**, *127*, 2846-2858.
(32) Strohmeier, M.; Stueber, D.; Grant, D. M. Accurate ^{13}C and ^{15}N chemical shift and ^{14}N quadrupolar coupling constant calculations in amino acid crystals: zwitterionic, hydrogen-bonded systems. *J. Phys. Chem. A* **2003**, *107*, 7629-7642.
(33) Socha, O.; Hodgkinson, P.; Widdifield, C. M.; Yates, J. R.; Dračínský, M. Exploring systematic discrepancies in DFT calculations of chlorine nuclear quadrupole couplings. *J. Phys. Chem. A* **2017**, *121*, 4103-4113.
(34) Alderman, D. W.; Sherwood, M. H.; Grant, D. M. Comparing, modeling, and assigning chemical-shift tensors in the cartesian, irreducible spherical, and icosahedral representations. *J. Magn. Reson.* **1993**, *101*, 188-197.
(35) António, C. T.; Évora, A. O. L.; Bernardes, C. E. S.; Diogo, H. P.; de Araujo, G. L. B.; Smith, P.; Byrn, S. R.; Fausto, R.; Minas da Piedade, M. E. Polymorphism in erlotinib hydrochloride: new insights into relative stability, thermal behavior, and structural differences of forms A and B. *Cryst. Growth Des.* **2023**, *23*, 7374-7384.
(36) Reddy, P.; Reddy, R.; Reddi, R. A novel hydrated form of erlotinib free base and a process for preparation of erlotinib hydrochloride polymorph form A substantially free of polymorph form B. EP2176241B1, 2007.
(37) Diem, M. *Modern Vibrational Spectroscopy and Micro-Spectroscopy : Theory, Instrumentation and Biomedical Applications*; John Wiley & Sons, Incorporated, 2015. DOI: https://doi.org/10.1002/9781118824924.part1.
